# Characterization of a Novel Phenol Hydroxylase in Indoles Biotranformation from a Strain *Arthrobacter* sp. W1

**DOI:** 10.1371/journal.pone.0044313

**Published:** 2012-09-13

**Authors:** Yuanyuan Qu, Shengnan Shi, Hao Zhou, Qiao Ma, Xinliang Li, Xuwang Zhang, Jiti Zhou

**Affiliations:** 1 Key Laboratory of Industrial Ecology and Environmental Engineering (Ministry of Education), School of Environmental Science and Technology, Dalian University of Technology, Dalian, China; 2 State Key Laboratory of Urban Water Resource and Environment, Harbin Institute of Technology, Harbin, China; Instituto Butantan, Brazil

## Abstract

**Background:**

Indigoids, as popular dyes, can be produced by microbial strains or enzymes catalysis. However, the new valuable products with their transformation mechanisms, especially inter-conversion among the intermediates and products have not been clearly identified yet. Therefore, it is necessary to investigate novel microbial catalytic processes for indigoids production systematically.

**Findings:**

A phenol hydroxylase gene cluster (4,606 bp) from *Arthrobacter* sp. W1 (PH_w1_) was obtained. This cluster contains six components in the order of *KLMNOP*, which exhibit relatively low sequence identities (37–72%) with known genes. It was suggested that indole and all the tested indole derivatives except for 3-methylindole were transformed to various substituted indigoid pigments, and the predominant color products derived from indoles were identified by spectrum analysis. One new purple product from indole, 2-(7-oxo-1H-indol-6(7H)-ylidene) indolin-3-one, should be proposed as the dimerization of isatin and 7-hydroxylindole at the C-2 and C-6 positions. Tunnel entrance and docking studies were used to predict the important amino acids for indoles biotransformation, which were further proved by site-directed mutagenesis.

**Conclusions/Significance:**

We showed that the phenol hydroxylase from genus *Arthrobacter* could transform indoles to indigoids with new chemical compounds being produced. Our work should show high insights into understanding the mechanism of indigoids bio-production.

## Introduction

Indigo, as one of the oldest dyes, is still used for printing and dyeing worldwide, which is primarily produced by chemical synthesis in modern textile industries [1]. However, researchers have been trying to seek more competitive and greener alternatives for commercial production, which has rejuvenated an interest in the microbial production of indigo [2]–[5]. Compared with indigo biosynthesis, there have been only sporadic reports on investigating the biotransformation of indole derivatives to form indigoid pigments [6], [7], which can be probably used as chief precursors in dyeing industries, and can also serve as anticancer compounds for therapeutic application [8], [9]. Recently, the intermediates derived from indole biotransformation have been identified such as isatic acid, 7-hydroxyindole, isoindigo, 2-oxindole, 6-hydroxyindole, dioxindole and indoxyl [10]–[13]. But the inter-conversion among these compounds needs further investigation and the new types of pigments from indoles should be purified and studied systematically.

During the history of microbial indigo production, the most representative study was performed by Ensley *et al*. in 1983, which proved that recombinant *Escherichia coli* expressing naphthalene dioxygenase (NDO) from *Pseudomonas putida* PpG7 could result in indigo formation [3]. Since then, a number of wild indigo-producing microorganisms induced by aromatic hydrocarbons and recombinant *E. coli* strains harboring monooxygenases (MOs) or dioxygenases (DOs) have been proved to transform indole to indigo or oxidized indoles [2]–[4], [11], [14]–[23]. The generally accepted pathway encoded by DOs is initiated by the oxidation of indole at the C-2 and C-3 positions to obtain *cis*-indole-2,3-dihydro-2,3-diol, which is then dehydrated to indoxyl, and the dimerization of two molecules of indoxyl leads to the production of indigo [3]. The transformation pathway encoded by MOs was firstly given by Mermod *et al*. in 1986 [24], which can be described as indole is directly hydroxylated at the C-3 position of the pyrrole ring forming indoxyl, and then undergoes dimerization to form indigo likewise. However, there has been no report on any kind of phenol hydroxylase (PH) in *Arthrobacter* strains as biocatalyst for indigoids biosynthesis from indoles. Therefore, the use of PH in the area of biocatalysis and fine chemical production needs to be thoroughly exploited.

As a member of bacterial multi component monoxygenases (BMMs), phenol hydroxylase (EC 1.14.13.7) was firstly identified in 1990 in *Pseudomonas* sp. CF600 responsible for phenol and (di)methylphenol degradation [25]–[27]. It was reported to catalyze the regiospecific hydroxylation of a number of substituted phenols at the *ortho* position with respect to the hydroxyl moiety [28]. Generally, PH is commonly composed of six polypeptides in the order of *KLMNOP*, which can be designated as PHK, PH(LNO)_2_, PHM and PHP [25], [29]. PHP is a NADH-oxidoreductase responsible for supplying electrons to the diiron cluster housed in the active site. The hydroxylase (200–255 kDa) is composed of three polypeptides (L, N, O) organized in a dimeric form (LNO)_2_. PHN is responsible for binding various substrates. PHM (10–16 kDa) is a regulatory protein, devoid of any cofactor or metal ion, which is essential for efficient catalysis. And PHK is responsible for assembling iron at the active site [30]. However, which residues play the key role in binding substrates of PH are still unclear. Therefore, it is essential to obtain information on the active site of the PHN, which will help improve our knowledge of enzyme-ligand interactions. It has been shown that molecular informatics technology is useful in revealing the configuration of the substrate-enzyme complex, and also exhibits significant abilities in predicting the catalytic potential of enzymes [31]–[33].

The aim of this study was to investigate the biotransformation of indoles to indigoids by a novel phenol hydroxylase from *Arthrobacter* sp. W1. Homology modeling and molecular docking were applied to analyze substrate specificity and interactions between indoles and PH. The color products were identified by HPLC-MS and NMR analysis. Our work should show high insights into the potential for commercial indigoids production by PH from genus *Arthrobacter*, which will pave the way to novel avenues in green chemistry.

## Results

### Cloning and expression of the PH gene cluster from strain W1

The primers (Raw TEST-F/Raw TEST-R) based on the conserved region of PH were used to amplify the genes encoding the large subunit of PH. A 685 bp homological gene was amplified from genomic DNA of strain W1. Thermal asymmetric interlaced PCR (TAIL-PCR, entails consecutive reactions with nested sequence-specific primers and a shorter arbitrary degenerate primer), a genome walking method, was adopted to amplify 5′ and 3′-terminal flanking regions [34]. After TAIL-PCR for the 5′-terminal flanking region, four ORFs (ORF1, ORF2, ORF3 and ORF4) were obtained. However, the length of the 5′-terminal flanking region obtained could not meet the requirements. Thus, the 5′-terminal flanking region was amplified again using special primers and then the full length was obtained. The 3′-terminal flanking region was amplified by three steps of TAIL-PCR, and the nucleotide sequence obtained was used to design the specific primers for further TAIL-PCR processes. After three steps of TAIL-PCR, two complete ORFs were obtained.

**Figure 1:**
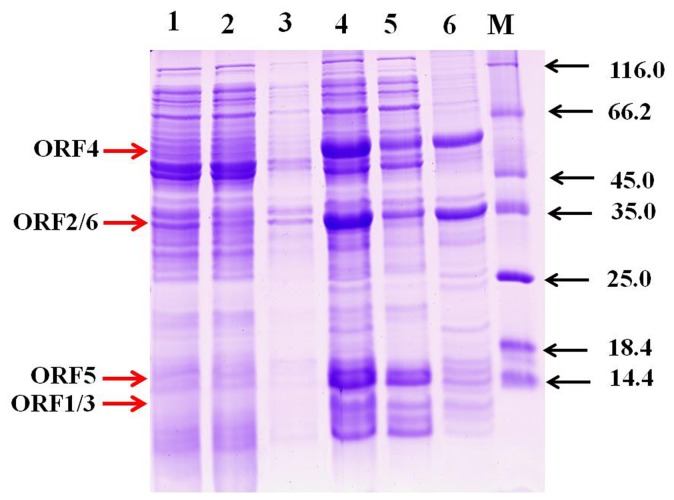
SDS-PAGE analysis of protein samples of PH__IND_ from *E. coli* BL21 (DE3). **Line 1.** Whole cells of pET-28a(+); **Line 2.** Cell extracts of pET-28a(+); **Line 3.** Precipitation of pET-28a(+); **Line 4.** Whole cells of strain PH__IND_; **Line 5.** Cell extracts of strain PH__IND_; **Line 6.** Precipitation of strain PH__IND_; **M.** Protein markers. Arrows show the positions of the six ORFs. **ORF1.** 10.4 kDa; **ORF2**. 37.6 kDa; **ORF3.** 10.5 kDa; **ORF4.** 59.2 kDa; **ORF5.** 13.5 kDa; **ORF6.** 38.6 kDa. SDS-PAGE was performed with 5% and 15% acrylamide concentrations for the concentrating and separating gels, respectively.

PH genes were successfully expressed (designated as PH__IND_) under the transcriptional control of a strong promoter T7, and the SDS-PAGE analysis revealed the presence of six ORFs for PH__IND_ (Figure 1). The effects of pH, salt concentration, temperature and metal ions on the activities of crude PH__IND_ were shown in Figure S1, which presented the optimal range of pH 6.0–8.0, salt concentration 0.5–1.0% and temperature 40–60°C. Under the optimal conditions, the *k_cat_* and *K_m_* of PH__IND_ with phenol were 2.137 s^−1^ and 5.429 µM, respectively. The *k_cat_/K_m_* of PH__IND_ (0.394 s^−1^/µM^−1^) was lower than that of phenol hydroxylase from *Pseudomonas stutzeri* OX1 [35].

### Operon organization and sequence alignment of PHs

The complete organization of the respective operon and conversed amino acids of various PHs in each multi component system were analyzed by BLAST. According to the results, all the PHs exhibited similar organization with six operons (Figure S2). Six ORFs from strain W1 showed relatively low sequence identities with those of several well-studied bacteria, such as *Ralstonia eutropha* E2, *Pseudomonas* sp. CF600, *Pseudomonas putida* H and *Comamonas testosteroni* R5 [27]. The most conserved ORF was the α subunit of oxygenase (*orf*4, N component in strain W1) with 64%–72% sequence identity. Whereas, the lowest sequence identity existed in the γ subunit of oxygenase (*orf*5, O component in strain W1), which showed 37%–42% identity with other corresponding components (Figure S2, Table S1).

As is well known, the N component (α subunit) harbors a non-heme carboxylate-bridged diiron center for catalysis and plays a key role in the selectivity of substrate. Therefore, it is essential to analyze and obtain the information of conserved residues in the N component. It was suggested that two typical EXRH motifs were found conserved at positions 138–141 and 233–236 (in strain *Pseudomonas* sp. OX1), which were assigned to the ligands of the catalytic diiron (Figure S2) [29].

### Biotransformation of indole and its derivatives by whole cells of strain PH__IND_

To investigate the substrate range of strain PH__IND_, *in vitro* assays were performed with indole and 14 kinds of derivatives. During the transformation process, the control group (host cells *E. coli* BL21 (DE3)) showed no activities toward all the substrates. Color formation could be observed with almost all the substrates (Table S2), however, the color exhibited differently from the same substrate compared with the previous literatures [6]–[8], [12], [20], [36]. Therefore, considering the type and position of the substituted group, 6 kinds of indole derivatives were selected for further investigation in this study. It was suggested that strain PH__IND_ was able to catalyze the formation of dyestuffs from indole, 4-, 5- and 7-methylindole, 4- and 7-chloroindole and 5-methoxyindole (Table 1). The substrates were classified into 4 groups, which were described in detail as follows.

**Table 1:** Characteristics of indigoids produced by whole cells of strain PH__IND_.

Substrate	Transformation yield (%)*	Products	NMR analysis of products
Indole	98.80	Indigo	^1^H NMR (400 MHz, *d_6_*-Me_2_SO) δ = 6.95 (t, 2H, J = 8 Hz), 7.33 (d, 2H, J = 8 Hz), 7.51 (t, 2H, J = 7.6 Hz), 7.61 (d, 2H, J = 8 Hz), 10.50 (s, 2H, NH)
		2-(7-oxo-1H-indol-6(7H)-ylidene) indolin-3-one	^1^H NMR (500 MHz, *d_6_*-Me_2_SO) δ = 6.27 (t, 1H, J = 1.9 Hz, H-2′), 6.81 (d, 1H, J = 9.5 Hz, H-4′), 7.07 (t, 1H, J_1_ = 7.4 Hz, J_2_ = 7.3 Hz, H-5), 7.23 (t, 1H, J = 2.5 Hz, H-3′), 7.44 (d, 1H, J = 7.9 Hz, H-7), 7.57 (t, 1H, J_1_ = 7.4 Hz, J_2_ = 7.8 Hz, H-6), 7.62 (d, 1H, J = 7.5 Hz, H-4), 7.95 (d, 1H, J = 9.4 Hz, H-5′), 11.99 (s, 1H, NH), 12.45 (s, 1H, NH′)
			^13^C NMR (100 MHz, *d*_6_-Me_2_SO): δ = 105.5 (C-2′), 112.2 (C-3a′), 114.2 (C-7), 118.7 (C-5′), 119.5 (C-4′), 119.8 (C-2), 122.7 (C-5), 124.6 (C-4), 127.6 (C-3′), 127.8 (C-7a′), 130.8 (C-6′), 136.9 (C-6), 140.1 (C-3a), 151.7 (C-7a), 175.8 (C-7′), 190.3 (C-3)
4-Methylindole	95.61	4,4′-dimethylindigo	^1^H NMR (400 MHz, *d_6_*-Me_2_SO) δ = 2.46 (s, 6H, CH_3_), 6.77 (d, 2H, J = 7.2 Hz), 6.95 (t, 2H, J = 8.4 Hz), 7.20 (d, 2H, J = 7.2 Hz), 11.04 (s, 2H, NH)
5-Methylindole	94.82	5,5′-dimethylindigo	^1^H NMR (400 MHz, *d_6_*-Me_2_SO) δ = 2.36 (s, 6H, CH_3_), 6.89 (d, 2H, J = 7.6 Hz),7.26 (dd, 2H, J_1_ = 7.6, J_2_ = 2 Hz), 7.70 (d, 2H, J = 8 Hz), 10.93 (s, 2H, NH)
7-Methylindole	89.34	7,7′-dimethylindigo	^1^H NMR (400 MHz, *d_6_*-Me_2_SO) δ = 2.46 (s, 6H, CH_3_), 7.00 (t, 2H, J = 8 Hz), 7.16 (d, 2H, J = 7.6 Hz), 7.52 (d, 2H, J = 8 Hz), 11.46 (s, 2H, NH)
4-Chloroindole	78.66	4,4′-dichloroindigo	^1^H NMR (400 MHz, *d_6_*-Me_2_SO) δ = 7.05 (d, 2H, J = 7.2 Hz), 7.38 (d, 2H, J = 7.2 Hz), 7.71 (dd, 2H, J_1_ = 8, J_2_ = 2.4 Hz), 11.46 (s, 2H, NH)
7-Chloroindole	78.89	5,5′-dichloroindigo	^1^H NMR (400 MHz, *d_6_*-Me_2_SO) δ = 7.00 (t, 2H, J = 7.6 Hz), 7.15 (d, 2H, J = 7.6 Hz), 7.52 (d, 2H, J = 8 Hz), 11.45 (s, 2H, NH)
5-Methoxyindole	91.25	5,5′-dimethoxyindigo	^1^H NMR (400 MHz, *d_6_*-Me_2_SO) δ = 3.77 (s, 6H, OCH_3_), 7.08 (d, 2H, J = 8 Hz),7.15 (dd, 2H, J_1_ = 8.8, J_2_ = 2.4 Hz), 7.26 (d, 2H, J = 8.8 Hz), 10.27 (s, 2H, NH)

* Transformation yield (%) was calculated as the reduced indoles concentration to the initial indoles concentration in the reaction systems.

**Figure 2:**
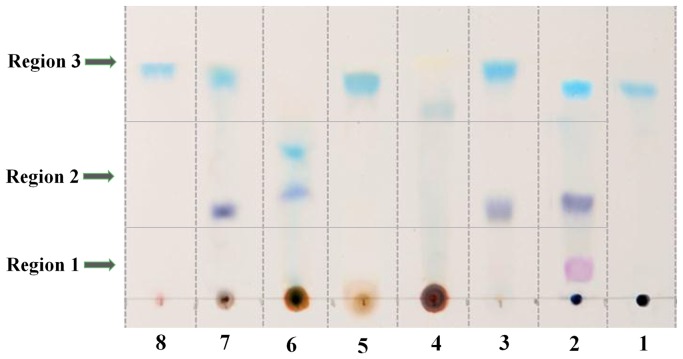
Identification of transformation products by TLC. The transformation samples were extracted with equal volume of ethyl acetate and concentrated by N_2_. 200 µL of the extracts were applied to the TLC plates (silica gel 60 F254), and then the TLC plates were resolved with a solvent mixture of dichloromethane-methanol (50∶1, v/v). The samples were designated as following: **1.** Indigo (standard); **2.** Products of indole transformation; **3.** Products of 4-methylindole transformation; **4.** Products of 5-methylindole transformation; **5.** Products of 7-methylindole transformation; **6.** Products of 5-methoxyindole transformation; **7.** Products of 4-chloroindole transformation; **8.** Products of 7-chloroindole transformation. The products with different *R_f_* values were indicated in three regions by the arrows.

Biotransformation of indole (group 1): When indole was incubated with strain PH__IND_, three colored products were observed by TLC with *R_f_* values of 0.70, 0.31 and 0.09, respectively (Figure 2, Sample 2). The molecular ion and retention time (*t_r_*) of the pink product (*R_f_* = 0.09) was at *m*/z 263 ([M^+^]) and 10.1 min (data not shown), which was identical to the authentic standard of indirubin. The HPLC retention time (*t_r_*) of the light blue product (*R_f_* = 0.70) was 9.6 min and its mass spectrum was primarily characterized by a molecular ion at *m*/z 263 ([M^+^]) (Figure S3), and its ^1^H NMR spectrum was consistent with the authentic standard of indigo (Table 1, Figure S4). According to the different polarity, the purple product (*R_f_* = 0.31) derived from indole was neither indigo nor indirubin, although the molecular mass was the same. The spectroscopic data of this product were listed in Table 1. ^1^H NMR and ^13^C NMR clearly showed an unsymmetrical structure of a pyrrole ring (hydrogen at C-2 and C-3 positions) connecting with a benzene ring (at C-6 and C-7 positions of the benzene ring) by a double bond. Therefore, the structure of purple product was proposed as 2-(7-oxo-1H-indol-6(7H)-ylidene) indolin-3-one, which was further certified by ^1^H-^1^H COSY, ^1^H-^13^C HSQC and ^1^H-^13^C HMBC (Figure S5).

Biotransformation of 4-, 5- and 7-methylindole (group 2): TLC analysis of 4-methylindole transformation products revealed the presence of two main color products with *R_f_* values of 0.76 and 0.31, respectively (Figure 2, Sample 3). HPLC-MS analysis of the light blue product showed a molecular ion of *m*/z 291 ([M^+^]) (Figure S3). The ^1^H NMR spectra exhibited a symmetric structure like indigo (Table 1, Figure S4). Thus, the light blue product from 4-methylindole was presumed to be 4,4′-dimethylindigo (Table 1). The other purple product was located in the same region with that derived from indole with a similar *R_f_* value (Figure 2, Sample 3, region 2), which implicated it should possess the similar structure to 2-(7-oxo-1H-indol-6(7H)-ylidene) indolin-3-one. However, only one color product derived from 5-methylindole was determined as 5,5′-dimethylindigo with *m*/z 291 ([M^+^]) and a *R_f_* value of 0.62 (Figure 2, Sample 4 and Figure S3). The ^1^H NMR shifts and assignments were shown in Table 1 and Figure S4. One color product formed from 7-methylindole (*R_f_* = 0.61) (Figure 2, Sample 5). HPLC-MS analysis of the product of 7-methylindole showed a molecular ion *m*/z of 291 ([M^+^]) (Figure S3). ^1^H NMR of the 7-methylindole product was exhibited in Table 1 and Figure S4. According to TLC, HPLC-MS and ^1^H NMR analysis, the single product of 7-methylindole was identified as 7,7′-dimethylindigo.

Biotransformation of 4- and 7-chloroindole (group 3): Biotransformation of 4-chloroindole yielded two color products with *R_f_* values of 0.74 and 0.30, respectively (Figure 2, Sample 7). The light blue color was identified as 4,4′-dichloroindigo (Table 1), and the other purple product was also presumed to be a 2-(7-oxo-1H-indol-6(7H)-ylidene) indolin-3-one like compound according to the analysis above. The mass spectra of the purified product showed a base peak at *m*/z 331 ([M^+^]) with an expected dichloride ratio of 9∶6∶1 for a peak at *m*/z 331, 332 and 333 (Figure S3). The ^1^H NMR analysis (Table 1 and Figure S4) was similar to that of 4,4′-dichloroindigo reported previously [12]. Biotransformation of 7-chloroindole led to the production of 7,7′-dichloroindigo as a single color compound, according to the *R_f_* value of 0.76 (Figure 2, Sample 8), HPLC-MS and ^1^H NMR analysis (Figure S3, Figure S4 and Table 1). The mass spectra were characterized by a molecular ion at *m*/z 331 ([M^+^]).

Biotransformation of 5-methoxyindole (group 4): The biotransformation of 5-methoxyindole yielded one main color product (*R_f_* = 0.51) (Figure 2, Sample 6). The mass spectrum of the product was determined by a molecular ion at *m*/z 321 ([M^+^]). The ^1^H NMR shifts and assignments were showed in Table 1 and Figure S4, which were consistent with the report by Guengerich *et al*. [8]. Thus, the color product was identified as 5, 5′-dimethoxyindigo. It was also found that the location of light blue and purple products from 5-methoxyindole showed in Sample 6 were a little different from Sample 2, Sample 3 and Sample 7 (Figure 2) due to the methoxy group at the C-5 position.

### Homology modeling of the catalytic domain of PH

**Figure 3:**
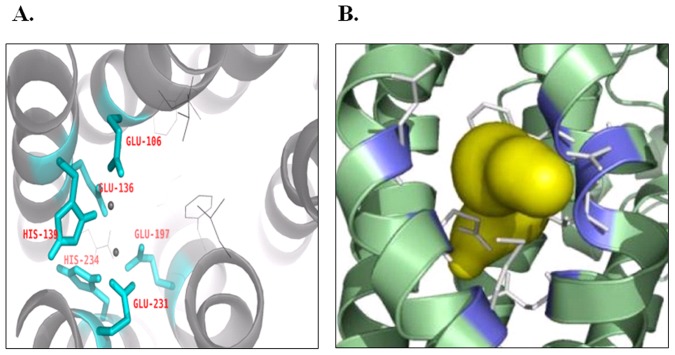
Homology modeling and identified substrate tunnel. **A.** The residues involved in coordinating dinuclear iron. These residues were labeled in cyan, and the dinuclear iron sites were shown in sphere; **B.** Tunnel identified in the homology modeling of PHN component from *Arthrobacter* sp. W1 using CAVER. The white sticks represent five formed residues, i.e. Thr-201, Asn-202, Phe-205, Glu-231 and Met-235. The tunnel is labeled as yellow surface and the residues formed entrance are in purple.

In order to explain the different conversion behavior of indole derives, three-dimensional structure of the N-component from PH_W1_ was constructed based on a homology template. Structural alignment of the PH_W1_ model to the template PH_OX1_ exhibited 0.16 Å of RMSD of the 488 residues, confirming that the fold was essentially the same. The quality validation of the model was performed by ERRAT, Verify_3D and PROCHECK analysis. As a typical feature of phenol hydroxylase, the dinuclear iron (II) sites were coordinated by four carboxylates and two histidines (Fe1 was coordinated by His-139, Glu-106 and Glu-136, Fe2 was coordinated by His-234, Glu-231 and Glu-197) (Figure 3A). In order to find the most direct route of small molecules entering the active site pocket, tunnel analysis of PHN_W1_ was performed by CAVER. The tunnel entrance was about 6.00 Å in diameter, which was located above the diiron center and formed by the side chains of Thr-201, Asn-202, Phe-205, Glu-231 and Met-235 (Figure 3B). Comparisons of the active sites in various oxygenases were shown in Table 2. It was suggested that the cavity of PHN_OX1_ is 587.1 Å^3^, which is larger than that of NDO, MMO and P450. As a homologous protein, PHN_W1_ (508.4 Å^3^) exhibited the similar cavity volume with PHN_OX1_. Therefore, taking entrance size and cavity volume into consideration, PH__IND_ seemed to be the efficient candidate for chemicals production.

**Table 2:** Comparisons of active sites in various oxygenases.

Form of iron	Enzyme*	PDB ID	Size of tunnel entrance (Å)	Volume of cavity (Å^3^)	Reference
Non-heme Fe (II)	PH *_Pseudomonas stutzeri_*_ OX1_	2INP	6.0	587.1***	(27)
	TMO *_Pseudomonas mendocina_*_ KR1_	3DHG	4.4	750.8***	(27)
	MMO *_Methylococcus capsulatus_*	1XU3	2.0	246.6***	(27)
	NDO *_Pseudomonas putida_*_ NCIB9816-4_	1EG9	4.6**	480.0	(37)
Heme Fe (II)	P450 2A6 _Human microsomal_	1Z10	<1.4	440.0	(12)

* PH: phenol hydroxylase; TMO: toluene 4-monooxygenase; MMO: methane monooxygenase;

NDO: naphthalene 1,2-dioxygenase.

** The distance of Val-260 CG1 and His-295 NE2.

*** Calculated by CASTp server.

**Figure 4:**
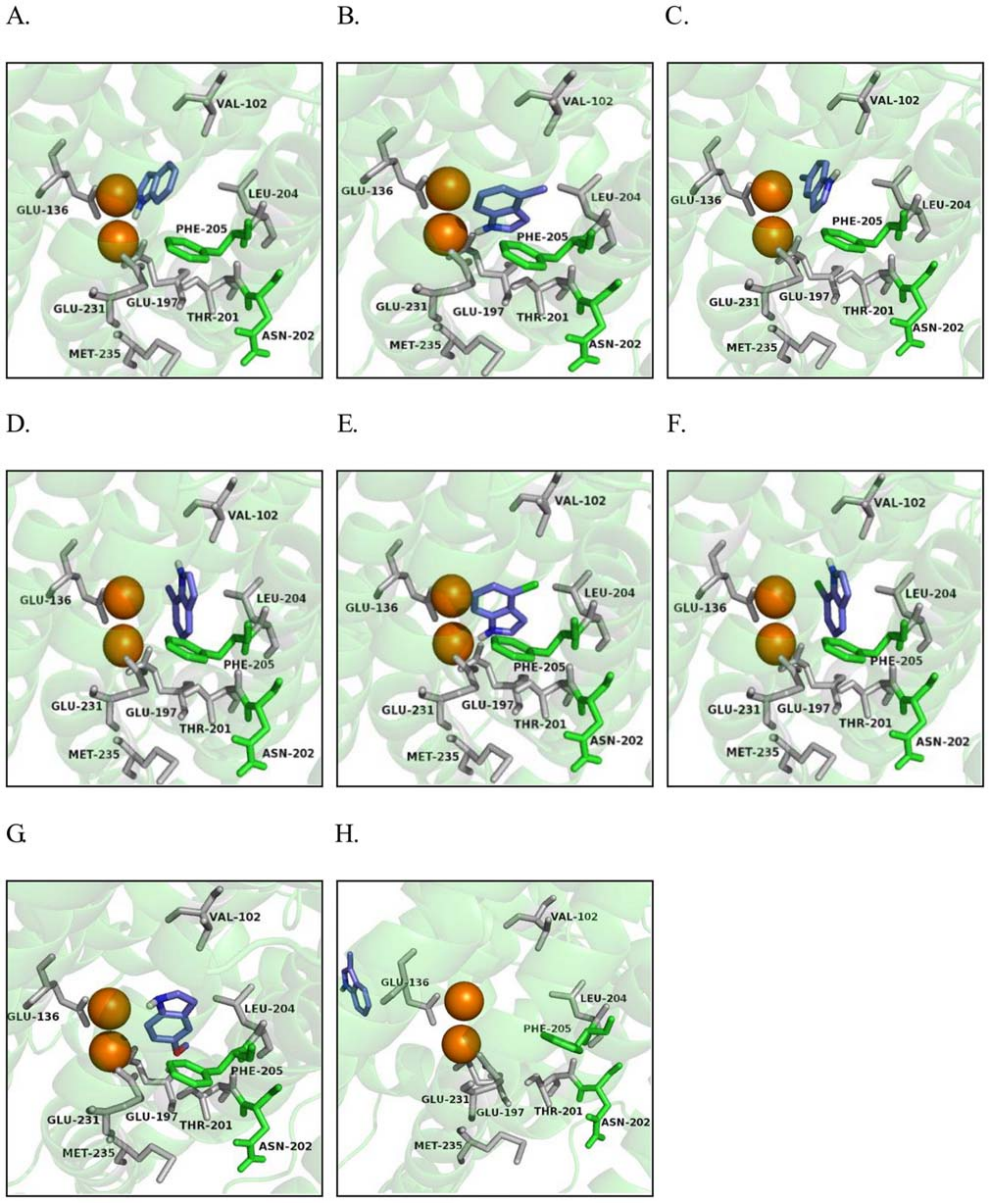
Interactions between PHN_W1_ component and indole derivatives. Orientations of docked indoles in the active site of PHN**_W1_** component: **A.** Indole; **B.** 4-Methylindole; **C.** 5-Methylindole; **D.** 7-Methylindole; **E.** 4-Chloroindole; **F.** 7-Chloroindole; **G.** 5-Methyoxyindole; **H.** 3-Methylindole. Atom designation: carbon atom, blue; hydrogen atom, white; chlorine atom, green; oxygen atom, red. Orange spheres represent for diiron, of which located above is designated as Fe1; green represent for tunnel entrance residues Asn-202 and Phe-205; other important residues are shown in grey.

With the modeled 3D structure of PHN_W1_, indole analogues were docked into the active site cavity. The most favorable binding conformations predicted by AutoDock vina were shown in Figure 4. The residues around the active site were shown in Table 3. Several interesting points could be implicated from the enzyme-substrate complex. Firstly, all the indole analogues could be classified into two-types: located far away from the diiron center (3-methylindole) (Figure 4H), and located near the diiron center (the other analogues) (Figure 4A–G). Secondly, the substrates, except for 3-methylindole, interacted with a number of residues including Leu-204, Phe-205 and Val-102 by hydrophobic and aromatic stacking, which might stabilize the enzyme-substrate complex. Meanwhile, several indole analogues could be stabilized by forming hydrogen bonds with the adjacent residues. 7-Chloroindole and 7-methylindole formed a hydrogen bond with Glu-136 (Figure 4D), 4-chloroindole and 4-methylindole formed hydrogen bond with Glu-231 (Figure 4B, E), while indole formed two hydrogen bonds with Glu-197 and Glu-136 (Figure 4A).

**Table 3:** Residues involved in binding different indoles.

Substrate	Residues within 4 Å
Indole	Val102 Leu105 Glu106 Ala109 Gln132 Glu136 Phe196 Glu197 Leu204
4-Methylindole	Glu106 His139 Gln143 Glu197 Thr201 Leu204 Phe205 Phe223 Ser227 Glu231 His234
5-Methylindole	Val102 Leu105 Glu106 Ala109 Phe177 Phe196 Glu197 Leu204 Glu231
7-Methylindole	Phe98 Val102 Glu106 Glu143 Phe196 Glu197 Thr201 Leu204 Phe205 Glu231
4-Chloroindole	Glu106 His139 Gln143 Glu197 Thr201 Leu204 Phe205 Met209 Phe223 Glu231 His234 Ser237
7-Chloroindole	Phe98 Val102 Glu106 Glu143 Phe196 Glu197 Thr201 Leu204 Phe205
5-Methyoxyindole	Phe98 Glu106 Gln143 Phe196 Glu197 Thr201 Leu204 Phe205 Phe208 Met209 Phe223 Ser 227 Glu231

The distance between C-7 of the benzene ring and Fe1of the active site was 2.96 Å for indole, 3.37 Å for 4-chloroindole, 3.39 Å for 4-methylindole, 3.49 Å for 5-methoxyindole, 4.43 Å for 5-methylindole, 3.72 Å for 7-methylindole and 3.70 Å for 7-chlorolindole. Whereas, C-7 of 3-methylindole was 17.5 Å away from Fe1of the active site, which indicated that it couldn't be transformed by strain PH__IND_. It was shown that indole and its derivatives were oriented in a different way (Figure 4). When indole was substituted at 4 or 5 positions, the orientations of substrates were adjusted to reduce the distance between C-7 and Fe1 of diiron (Figure 4B, 4E, 4G) [12]. Consequently, it proved that indole and its derivatives could be catalyzed by strain PH__IND_ to some extent, and also the C-7 position seemed more easily to be attacked by strain PH__IND_ than other C atoms in the six-membered ring.

### Critical residues identification by site-directed mutation

In order to verify the importance of gating and iron binding residues identified by homology modeling, Asn-202 and His-139 were taken for mutagenesis studies, which were located at the entrance of active site pocket and diiron center (Fe1), respectively. The Asn-202 was mutated to Phe-202 with a bulky side chain, which can reduce the size of entrance from 6.00 Å to 4.47 Å. Meanwhile, the His-139 was mutated to Ala-139, which could not coordinate with iron. SDS-PAGE of crude cell extracts from two mutants was shown in Figure S6. It was suggested that both mutants lost the catalytic activity of transforming indole to indigo (Figure S7), which indicated that Asn-202 and His-139 were the critical residues for indole transformation.

## Discussion

In this study, we designed the experiments to show that PH obtained from *Arthrobacter* sp. W1 can be used to produce dyestuffs from indole and its derivatives. The indigo productivity of 44.75 mg L^−1^ h^−1^ by whole cells of strain PH__IND_ was obtained in this study. As previously reported, a recombinant *E. coil* OST3410 carrying the PH gene from *Acinetobacter* sp. ST-550 produced 52 µg mL^−1^ of indigo in the presence of diphenylmethane at 24 h [14]. Cultures of *E. coli* JM101 that expressed HbpA_ind_ during growth in LB medium had an indigo productivity of 5 mg L^−1^ h^−1^ [20]. Recombinant *E. coli* HB101 expressed the naphthalene dioxygenase gene produced 1 mg L^−1^ h^−1^ of indigo [3]. Therefore, strain PH__IND_ seemed to be more efficient than other recombinant strains. However, the indigo yield is not the only standard to evaluate the function of indigo-producing microorganisms, because the transformation depends on various variables, such as inducers, solvents and culture conditions. As reported previously, *E. coli* strains expressing mPH from *Pseudomonas* sp. KL28 and KL33 could catalyze the production of dyestuffs and hydroxyl indoles from indole derivatives [6]. In comparison with mPH_KL28_ and mPH_KL33_, strain PH__IND_ can oxidize indole derivatives to a larger library of new and complex indigoids. The recombinant *E. coli* expressing various enzymes responsible for indoles transformation were summarized in Table S2. By contrast, strain PH__IND_ exhibited strong catalytic function, which could hydroxylate 14 kinds of indoles.

As for indole transformation by strain PH__IND_, three color products (indigo, indirubin and 2-(7-oxo-1H-indol-6(7H)-ylidene) indolin-3-one) were identified (Figure 2). 2-(7-oxo-1H-indol-6(7H)-ylidene) indolin-3-one appeared to be a novel pigment, because no such structure was reported by oxidizing indole using any kind of oxygenase. In the recent reports, some novel indigoid pigments have been identified and produced, which should be used as dyestuffs and be of therapeutic values [11], [12]. Due to their similar chemical structures, this new product would be potential as the precursor for both dyeing and pharmaceutical production. The similar structure was also identified by the mutant (N297Q/I300V) of cytochrome P450 2A6, which oxidized 4-benzyloxy(OBzl) indole to form a color product (4-OBzl-2-(4′-OBzl-1′,7′-dihydro-7′-oxo-6′H-indol-6′-ylidene) indolin-3-one) [12].

In order to investigate the relationship between the function (substrate specificity) and structure (of active site and substrate-enzyme complex), molecular simulation was performed. The surface residues involved in the formation of tunnel, which named Thr-201, Asn-202, Phe-205, Glu-231 and Met-235, were conserved among different PHs. The pore diameter (∼6.00 Å) was large enough to accommodate indole and substituted indoles (Table 2). However, the analogous pore was absent in some crystal structures of BMMs due to the shifting of Asn-214 (Asn-202 in PHN_W1_), which suggested that the size of substrate entrance to the diiron center was the determinant for substrate selectivity of BMMs [32]. In the previous literature, cytochrome P450 2A6 was modified by random and site-directed mutagenesis and the mutant I300V could transform bulky substituted indoles [12]. The mutant provides an additional 83 Å^3^ space of active site, which is large enough to allow the bulky substituted indoles binding in a suitable conformation [12]. As shown in Table 2, the cavity of PH__OX1_ is 587.1 Å^3^, which is larger than that of NDO, MMO and P450, which suggested potential versatile substrate spectra of PH__OX1_. Therefore, taking entrance size and cavity volume for consideration, PH is an efficient candidate for chemicals production.

However, not only the entrance size and cavity volume of active pocket, but also the suitable orientation of substrate would affect the specificity of enzyme. To our best knowledge, there is no crystal structure of PH-indole (or indole derivatives) complex, so docking studies can be applied to obtain the binding information of different substrates. Firstly, the lowest energy binding conformation of 3-methylindole was significantly different from others, which indicated that it was hard to be transformed. Secondly, the bound mode depended on the position and type of the substituent, which meant the substituent on different position of indole should affect the affinity between ligands and protein. It was previously reported that when indole was docked into the active cavity of naphthalene 1,2-dioxygenase (NDO), indole was oriented as the five-membered ring pointing inward, while the six-membered ring pointing towards the entrance of the substrate channel [37]. However, both five-membered ring and six-membered ring in this study were oriented in the same way to the diiron active site, which were different from the binding mode of NDO. All the docking analyses can supply useful information to predict the potential performance of PH__IND_ for indoles oxidation.

Indole belongs to heteroaromatic with π-election densities on carbon atoms of the pyrrole ring greater than those of the benzene ring, and it maintains an unbroken benzene ring and bears a positive charge on nitrogen and a negative charge on the C-3 atom, which means that C-3 is highly reactive toward electrophilic reagents [1]. All in all, the potential position of PH attacking substrates is determined not only by indole molecular characteristics (e.g. charge, substitute group and electronic density), but also by the distance between C atoms of the six-membered ring (especially C-7) and the active site Fe1. The site-directed mutagenesis can explain well that the substrate tunnel and residue involved in coordinating iron are truly essential for catalysis.

It is no accident that the purple compound is found from transformation of indole, 4-methylindole, 4-cholorlindole and 5-methoxyindole. It reveals that C-7 of the six-membered ring locates the nearest to the active site Fe1, whereas other C atoms are far away from the active site. As previously reported, C-3 of the five-membered ring was the most active position, which could be firstly attacked by the oxygenase [3], [11]. In addition, the position and type of substituted group is a secondary factor to be considered. We presume that the distance between C-7 and Fe1 is the predominant factor to form the purple compounds. According to the docking studies, we find that although the position of the methyl group and methoxy group are both at 5 positions, there is no purple product formed during the process of 5-methylindole transformation. This can be explained by the orientation of the two molecules are distinctly different from each other (Figure 4). Also, the distances between C-7 and Fe1 for 5-methylindole and 5-methoxyindole are 4.43 Å and 3.49 Å, respectively. Considering both the orientation tendency and the distance between C-7 and Fe1, 5-methylindole should not be transformed to a purple product.

**Figure 5:**
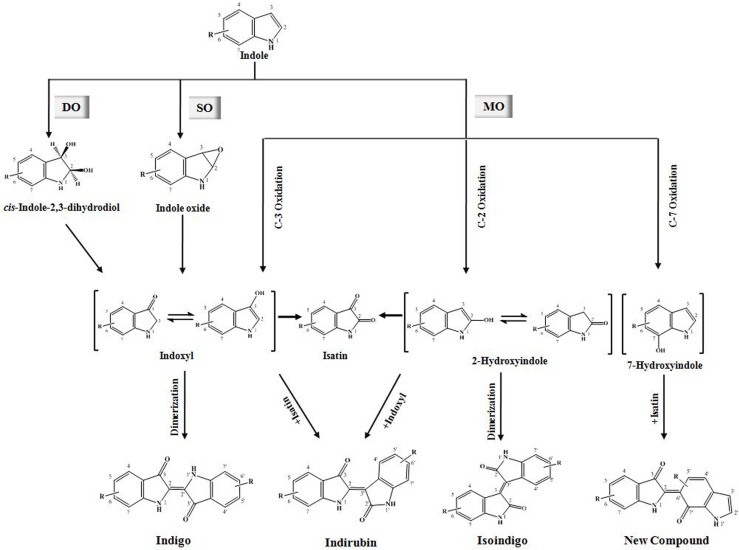
Summary of transformation pathways of indole by various oxygenases. **DO.** dioxygenase; **SO.** styrene oxygenase; **MO.** monooxygenase. R represents for substitute group i.e. methyl-, chloro-, methyoxy-, etc. The pathways catalyzed by strain PH__IND_ could be proposed by C-3 oxidation and C-7 oxidation pathways. The indole is firstly hydroxylated at the C-3 positions to form indoxyl (3-hydroxyindole) by strain PH__IND_, which undergoes further oxidation to form isatin as well as the indigoids precursors. Finally, two molecules of indoxyl polymerize to form indigo, and indoxyl can form indirubin with isatin. And the new compound is formed by 7-hydroxyindole and isatin.

According to the analysis mentioned above, the overview of the proposed pathways by various oxygenases is depicted in Figure 5. Firstly, indole can be attacked at C-2 and C-3 positions to form *cis*-indole-2,3-dihydrodiol and indole oxide by dioxygenase and styrene oxygenase, respectively [3], [22]. Then it undergoes the C-3 oxidation pathway, in which indigo and indirubin should be formed as the main color products. From Figure 5, it is obvious that the versatile products should be contributed to monooxygenase (including P450). There are three pathways by different MOs, which are named C-3, C-2 and C-7 oxidation pathways. From the results of this study and previous reports, C-3 often occurs with the C-2 and C-7 oxidation pathways. In other words, most MOs can hydroxylate indoles at the C-3 position, which leads to form indigo and indirubin. Whereas, some MOs can attack both C-3 and C-2 positions, thus the products should be composed of indigo, indirubin and isoindigo [2], [5], [11], [19], [24]. In the previous studies, both C-2 and C-3 oxidation pathways are generally accepted and well studied [8], [23], [38]. The C-7 oxidation pathway is previously reported by another *Pseudomonas* PH yielding 7-hydrxyindole from indole [6] and by P450, which produced one new product, i.e. 4-OBzl-2-(4′-OBzl-1′,7′-dihydro-7′-oxo-6′H-indol-6′-ylidene)indolin-3-one from 4-OBzl-indole [12]. Other than this, this is the first report on the formation of such compound.

In conclusion, we identified the phenol hydroxylase gene cluster responsible for indigoids bio-production and the color transformation products were indentified. Meanwhile, the catalytic mechanisms of PH__IND_ were proposed as the dimerization of isatin and 7-hydroxylindole at the C-2 and C-6 positions leading to form a novel purple product. Also, docking studies supported the experiments well and predicted some important residues, which is conductive to perform site-directed mutagenesis to expand the substrate specificities.

## Materials and Methods

### Chemicals

Indole, indirubin, indigo, 3-, 4-, 5- and 7-methylindole, 5-methoxyindole, 4- and 7-chloroindole, kanamycin and isopropyl-β-D-thiogalactopyranoside (IPTG) were purchased from J&K Scientific Ltd. (China). All other reagents and solvents were obtained from general commercial suppliers and used without further purification.

Genome Walking Kit, Mini BEST Plasmid and DNA Purification Kit, DNA Ligation Kit, and Primer STAR® HS DNA Polymerase, *Sca* I, *Hin*d III, *Aat* II and *Sal* I were all purchased from TaKaRa Co. Ltd. (Dalian, China), and used according to the instructions of the manufacturer.

### Bacterial strains, plasmids and growth conditions

The bacterial strains and plasmids used in this study were listed in Table 4. The vector plasmids, pMD18-T and pET-28a(+), were purchased from TaKaRa Co. Ltd. (Dalian, China) and Novagen (USA), respectively. pET-28a(+) containing PH*KLMNOP* cloned from strain W1 was constructed in this study. Strain W1 was cultured as previously described [39]. *E. coli* strains were grown in lysogeny broth (LB) medium containing kanamycin (Kan, 30 µg/mL) at 37°C and 150 r/min. For PH gene expression, *E. coli* BL21 (DE3) harboring the appropriate pET-28a(+) hybrid plasmid was induced by addition of IPTG (1 mM).

**Table 4:** Bacterial strains and plasmids used in this study.

Strains or plasmids	Characteristics	Source
**Strains**		
*Arthrobacter* sp. W1	Wild type, CGMCC 4763, phenol^+^, indole^+^, benzoic acid^+^, salicylic acid^+^, aniline^+^, catechol^+^, *o* (*m*, *p*)-cresol^+^, hydroquinone^+^, *o* (*m, p*)-aminophenol^+^, *m* (*p*)-nitroaniline^+^	(39)
*E. coli* JM109	*recA*1, *endA*1, *gyrA*96, *thi*-1, *hsdR*17 (*r*_k_^−^ *m*_k_^+^), *e*14^−^ (*mcrA* ^−^), *supE*44, *relA*1, Δ (*lac-proAB*)/F′ [*traD*36, *proAB* ^+^, *lac I* ^q^, *lacZ*ΔM15]	TaKaRa
*E. coli* BL21 (DE3)	F^−^, *omp*T, *hsd*S_B_ (*r*_B_^−^ *m*_B_^−^), *gal* (λ *c I* 857, *ind*1, *Sam*7, *nin*5, *lac*UV5-T7*gene*1), *dcm* (DE3)	TaKaRa
**Plasmids**		
pMD18-T	Vector used for cloning	TaKaRa
pET-28a (+)	Vector used for expressing	Novagan
pET-28a (+)/PH	The recombinant plasmid containing PH genes	This study

### Cloning, sequence analysis, and expression of PH gene cluster

DNA manipulations were carried out according to standard operating procedures [40]. The primers used in this study were listed in Table S3. Genomic DNA of strain W1 was extracted using Mini BEST Bacterial Genomic DNA Extraction Kit (TaKaRa Co. Ltd. Dalian, China). The primers (Raw TEST-F/Raw TEST-R) based on the conserved region of PH were used to amplify the target genes. Conditions for PCR were as follows: 98°C for 1 min, followed by 30 cycles of 98°C for 10 s, 55°C for 15 s, 72°C for 30 s, and a final extension step of 10 min at 72°C. The PCR products were recovered by TaKaRa Agarose Gel DNA Purification Kit Ver. 2.0 and sequenced by TaKaRa Co. Ltd. (Dalian, China). Three nested PCR procedures were applied to amplify the upstream and downstream regions of PH by TAIL-PCR using TaKaRa Genome Walking Kit (TaKaRa Co. Ltd. Dalian, China). The fragments containing the entire coding sequences were isolated and cloned into the compatible sites of pMD18-T simple vector for sequencing.

After sequencing, the genes were subcloned into the pET-28a(+) by *Sac* I and *Hin*d III yielding the recombined plasmid pET-28a(+)/PH. Subsequently, the resulting plasmids were introduced into *E. coli* BL21 (DE3) for expression, and the recombinant strain harboring the PH genes was designated as PH__IND_.

Analysis of potential ORFs and comparison of amino acid sequences (or nucleotide sequences) were performed with the ORF finder and BLAST programs on the National Center for Biotechnology Information website. Multiple-sequence alignment was performed by Clustal ×1.8.

### Construction of site-directed mutants

Mutants were produced by site-directed mutagenesis by the method of Tang *et al* [41]. The mutants with mutation position Asn-202 and His-139 were obtained using plasmid pET-28a(+)/PH__IND_ as the template by three-step PCR. The mutagenic primers were listed in Table S4. The resulting PCR products (1.1 kb) were completely sequenced, digested with *Aat* II and *Sal* I, and cloned into the plasmid pET-28a(+)/PH digested with the same enzymes. The resulting plasmid were designated as pET-28a(+)/PH -Asn-202 and pET-28a(+)/PH -His-139. For protein expression, the recombinant plasmids were introduced into *E. coli* BL21 (DE3), and the recombinant strains were designated as PH__IND_-Asn-202 and PH__IND_-His-139, respectively.

### Enzyme assay

Enzyme activities of the cell extracts were determined at room temperature by UV/Vis spectrophotometer. The cell pellets were disrupted by sonication (225 W at 4°C for 30 min, Ultrasonic processor CPX 750), and then the cell debris was removed by centrifugation at 22000 r/min for 20 min at 4°C. The supernatant was immediately used for the assays of enzyme activities. For phenol hydroxylase activity, assay mixtures contained 50 mM Tris-HCl (pH 8.0), 2.5 µM NADH, 0.02 mg protein and 200 mg/L phenol. Each assay was started with the addition of NADH to the reaction mixture containing fresh crude extracts. Activity of phenol hydroxylase was measured by the consumption of NADH at 340 nm. One unit of activity was defined as the amount of enzyme that caused the oxidation of 1 µM NADH per min in the presence of phenol. The kinetic parameters (*k_cat_*, *K_m_* and *k_cat_/K_m_*) were determined using whole cells as described previously [35].

The effects of pH (50 mM Na_2_HPO_4_/NaH_2_PO_4_ (pH 6.0–7.0) and Tris-HCl (pH 8.0–11.0)), temperature (20–70°C), phenol concentration (50–3000 µM), salt concentration (0.5%–5%) and metal ions on enzyme activities were determined.

The proteins of strain PH__IND_ and its mutants were subjected to SDS-polyacrylamide gel electrophoresis (SDS-PAGE) with 5% and 15% acrylamide for the concentrating and separating gels, respectively.

### Biotransformation and products identification

All biotransformation reactions were performed in 50-mL flasks at 30°C and 150 r/min. The control group was set using host cells of *E. coli* BL21 (DE3). For whole cell preparation, cells were harvested by centrifugation (8000 r/min, 15 min), washed with phosphate sodium buffer (0.1 M, pH 7.0), and resuspended in the same buffer to OD_600_ = 2.0. The reaction mixtures consisted of 20 mL cell suspensions, 1 mM glucose and 0.2 g/L indole derivatives using dimethyl sulfoxide (DMF) as a cosolvent. After completion of the reactions (20 h), the mixtures were extracted with an equal volume of ethyl acetate.

For thin layer chromatography (TLC) analysis, the ethyl acetate samples were removed with stream nitrogen. The resulting dried pigment cells were dissolved in dichloromethane to a volume of 500 µL. The thickness of the TLC plate was 0.15∼0.2 mm (silica gel 60 F254), on which 200 µL of sample was added by several times, and then the TLC plates were resolved with a solvent mixture of dichloromethane-methanol (50∶1, v/v).

Following TLC separation of the indigoid pigments described above, the separated pigment-containing samples were isolated from the TLC plate and the pigments were extracted from the silica matrix with dichloromethane. The solvents were evaporated, and the pigments were suspended in *d*_6_-Me_2_SO for liquid chromatography and mass spectrometry (HPLC-MS) analysis (Hewlett Packard 1100 MSD, America). The HPLC was operated as: RX-C18 column (150 mm×2.1 mm Agilent, CA, USA); 65% (v/v) CH_3_OH (in H_2_O containing 0.1% formic acid) for 10 min, followed by a 65–75% (v/v) CH_3_OH linear gradient over 20 min; flow rate, 0.2 mL/min; column temperature, 25°C; UV detection, 242 nm. MS was equipped with a standard API-1 atmospheric pressure chemical ionization (APCI) source in the positive or negative ion mode. N_2_ was used as a sheath gas (50 p.s.i.), vaporizer temperature was set to 350°C, and the corona current was maintained at 5 µA. The capillary was set at 220°C and 25 V (or −25 V in the negative mode). The tube lens voltage was set to 80 V (or to −96 V in the negative mode). The collision-induced dissociation was set to −30 V in tandem mass spectrometry experiments.

All the NMR experiments were performed on a Varian INOVA-500 MHz spectrometer at 298 K (Bruker Avance 500 Hz, Switzerland). Detailed structural information for purple product from indole was obtained by three separate two-dimensional (^1^H-^1^H COSY, ^1^H-^13^C HSQC and ^1^H-^13^C HMBC) NMR analysis.

### Modeling of PHN_W1_ and substrates docking

The initial three-dimensional model of the PHN**_W1_** component was built by homology-modeling method [42]. The crystal structure of PH from *Pseudomonas* sp. OX1 solved by Sazinsky *et al*. (PDB ID: 2INP) was chosen as the template based on the result of protein sequence blast (BLASTP) [29]. The general simulation method and model quality validation were similar to the previous report [42]. Modeller 9v8 was used to construct the homology modeling of PHN **_W1_** containing diiron atoms. To find interactions between active site and protein surface, the most possible tunnel was identified using CAVER [43].

Molecular docking studies performed by AutoDock Vina 1.1.1 were used to identify the important interactions between PHN and indole derivatives [44]. The coordinate files of ligands were prepared by PRODRG server. Cα of Leu105 was defined as the center of the 40×40×40 grid box. PyMOL was used to visualize the docking results and measure the distance between two atoms.

### Nucleotide sequence accession number

The nucleotide sequence of the phenol hydroxylase gene cluster (4606 bp) of *Arthrobacter* sp. W1 has been submitted to GenBank with the accession number FJ610336.

## Supporting Information

Figure S1 **Characteristics of crude PH__IND_.** A. The effects of pH on the enzyme activity. Assay mixtures contained different pH (5.0–11.0), 2.5 µM NADH, 0.02 mg protein and 200 mg/L phenol at 20°C; **B.** The effects of salt concentration on the enzyme activity. Assay mixtures contained 50 mM Tris-HCl (pH 8.0), 2.5 µM NADH, 0.02 mg protein and different concentrations of NaCl (0.5–5%) at 20°C; **C.** The effects of metal ions on the enzyme activity. Assay mixtures contained 50 mM Tris-HCl (pH 8.0), 2.5 µM NADH, 0.02 mg protein and 200 mg/L phenol at 20°C with 1 mM of each metal ions; **D.** The effects of temperature on the enzyme activity. Assay mixtures contained 50 mM Tris-HCl (pH 8.0), 2.5 µM NADH, 0.02 mg protein and 200 mg/L phenol at different temperature (20–70°C).
(PDF)

Figure S2 **Gene cluster analysis and primary structure alignment of PHN component.** **A.** PH gene cluster from strain W1 and related strains *Comamonas testosteroni* R5, *Ralstoniaeutropha* E2, *Pseudomonas* sp. CF600, *Pseudomonas putida* H. **B.** Structural alignment of primary structure of PHNs from *Pseudomonas* sp. OX1, *Pseudomonas* sp. CF600, *Acinetobacter radioresistens* S13, *Arthrobacter* sp. W1, *Ralstonia eutropha* E2 and *Bacillus thermoleovorans* sp. A2. The top line shows the secondary structure of PHN of *Pseudomonas* sp. OX1. The conserved residues are shown in red background.
(PDF)

Figure S3 **Mass spectra of purified indigoid products formed from indoles by strain PH__IND_.** **A.** Products of indole transformation; **B.** Products of 4-methylindole transformation; **C.** Products of 5-methylindole transformation; **D.** Products of 7-methylindole transformation; **E.** Products of 4-chloroindole transformation; **F.** Products of 7-chloroindole transformation; **G.** Products of 5-methoxyindole transformation. HPLC-MS conditions were as follows: HPLC, 65% (v/v) CH_3_OH (in H_2_O containing 0.1% formic acid) for 10 min, followed by a 65–75% (v/v) CH_3_OH linear gradient over 20 min; MS was equipped with a standard API-1 atmospheric pressure chemical ionization (APCI) source in the positive or negative ion mode. N_2_ was used as a sheath gas (50 p.s.i.), vaporizer temperature was set to 350°C, and the corona current was maintained at 5 µA. The capillary was set at 220°C and 25 V (or −25 V in the negative mode). The tube lens voltage was set to 80 V (or to −96 V in the negative mode). The collision-induced dissociation was set to −30 V in tandem mass spectrometry experiments.
(PDF)

Figure S4 **^1^H NMR spectra of purified indigoid products formed from indoles by strain PH__IND_.** **A.** Products of indole transformation; **B.** Products of 4-methylindole transformation; **C.** Products of 5-methylindole transformation; **D.** Products of 7-methylindole transformation; **E.** Products of 4-chloroindole transformation; **F.** Products of 7-chloroindole transformation; **G.** Products of 5-methoxyindole transformation. ^1^H NMR was carried out at 298 K with Bruker Avance II 400 M and 400 MHz instrument and the samples were dissolved in *d*_−6_ Me_2_SO.
(PDF)

Figure S5 **Identification of new purple product derived from indole.** **A.** Mass spectra; **B.** ^1^H NMR spectra; **C.** ^13^C NMR spectra; **D.** ^1^H-^1^H COSY; **E.** ^1^H-^13^C HSQC; **F.** ^1^H-^13^C HMBC. The conditions for each spectrum were the same with those described above.
(PDF)

Figure S6 **SDS-PAGE analysis of protein samples of strain PH__IND_ and its mutants.** **Line 1.** Cell extracts of strain PH__IND_; **Line 2.** Cell extracts of strain PH__IND_-Asn-202; **Line 3.** Cell extracts of strain PH__IND_-His-139; **M.** Protein markers. Arrows show the positions of the six ORFs. **ORF1.** 10.4 kDa; **ORF2.** 37.6 kDa; **ORF3.** 10.5 kDa; **ORF4.** 59.2 kDa; **ORF5.** 13.5 kDa; **ORF6.** 38.6 kDa. SDS-PAGE was performed on 5% and 15% acrylamide concentrations for the concentrating and separating gels, respectively.
(PDF)

Figure S7 **Biotransformation of indole by strain PH__IND_ and its mutans.** **A.** Indole biotransformation by strain PH__IND_; **B.** Indole biotransformation by strain PH__IND_-Asn-202; **C.** Indole biotransformation by strain PH__IND_-His-139. The left bottles were the control groups at 0 h; the right bottles were the test groups at 12 h.
(PDF)

Table S1 **Amino acid identity between PH_W1_ and other binuclear iron hydroxylases.**
(PDF)

Table S2 **Production of dyestuffs from indole derivatives by** ***Escherichia coli*** **expressing different oxygenases.**
(PDF)

Table S3 **Oligonucleotide primers used in this study.**
(PDF)

Table S4 **Primers used for site-directed mutagenesis.**
(PDF)
