# Correction: Characterization of a Novel Phenol Hydroxylase in Indoles Biotransformation from a Strain *Arthrobacter* sp. W1

**DOI:** 10.1371/annotation/087266ef-a19f-4224-a7c2-119d1b363331

**Published:** 2012-10-31

**Authors:** Yuanyuan Qu, Shengnan Shi, Hao Zhou, Qiao Ma, Xinliang Li, Xuwang Zhang, Jiti Zhou

The word "Biotransformation" is misspelled in the article title. The correct title is: Characterization of a Novel Phenol Hydroxylase in Indoles Biotransformation from a Strain *Arthrobacter* sp. W1.

The correct citation is: Qu Y, Shi S, Zhou H, Ma Q, Li X, et al. (2012) Characterization of a Novel Phenol Hydroxylase in Indoles Biotransformation from a Strain *Arthrobacter* sp. W1. PLoS ONE 7(9): e44313. doi:10.1371/journal.pone.0044313 

