# Supplementary material for: Characterization of a Novel Phenol Hydroxylase in Indoles Biotranformation from a Strain Arthrobacter sp. W1
Source: PLoS One. 2012 Sep 13;7(9):e44313. doi: 10.1371/journal.pone.0044313 (PMC3441600; doi:10.1371/journal.pone.0044313)
Supplement: Figure S1 — Characteristics of crude PH_IND. A. The effects of pH on the enzyme activity. Assay mixtures contained different pH (5.0–11.0), 2.5 µM NADH, 0.02 mg protein and 200 mg/L phenol at 20°C; B. The effects of salt concentration on the enzyme activity. Assay mixtures contained 50 mM Tris-HCl (pH 8.0), 2.5 µM NADH, 0.02 mg protein and different concentrations of NaCl (0.5–5%) at 20°C; C. The effects of metal ions on the enzyme activity. Assay mixtures contained 50 mM Tris-HCl (pH 8.0), 2.5 µM NADH, 0.02 mg protein and 200 mg/L phenol at 20°C with 1 mM of each metal ions; D. The effects of temperature on the enzyme activity. Assay mixtures contained 50 mM Tris-HCl (pH 8.0), 2.5 µM NADH, 0.02 mg protein and 200 mg/L phenol at different temperature (20–70°C). (PDF) [file pone.0044313.s001.pdf]

A.

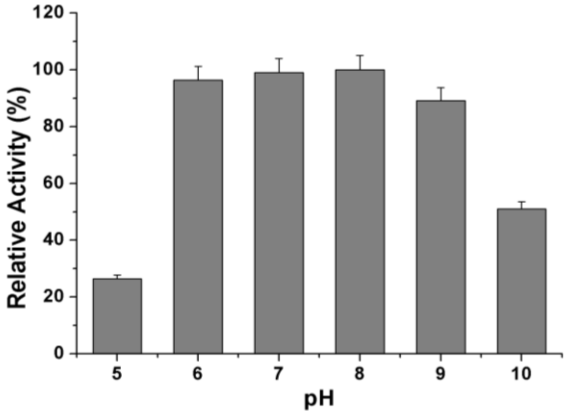

B.

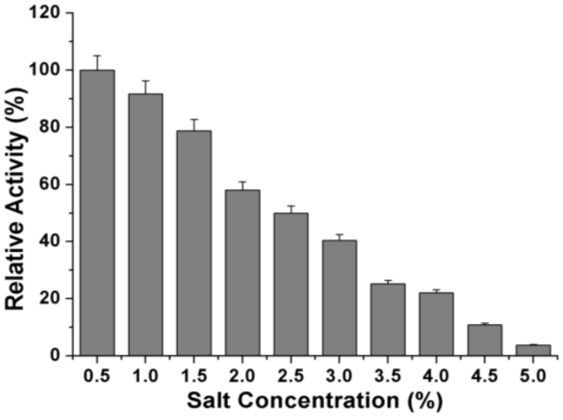

C.

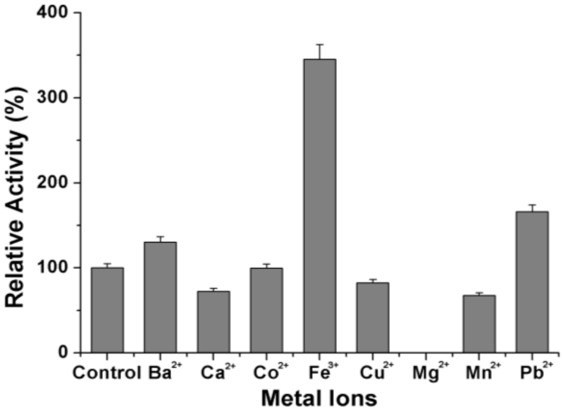

D.

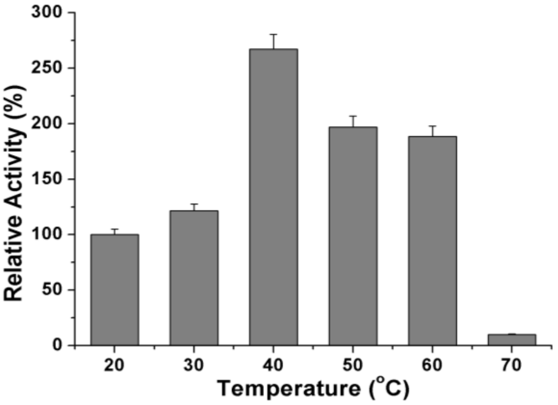

Figure S1. Characteristics of crude PH$_{_IND}$. A. The effects of pH on the enzyme activity. Assay mixtures contained different pH (5.0-11.0), 2.5 μM NADH, 0.02 mg protein and 200 mg/L phenol at 20 ^{o}C; **B.** The effects of salt concentration on the enzyme activity. Assay mixtures contained 50 mM Tris-HCl (pH 8.0), 2.5 μM NADH, 0.02 mg protein and different concentrations of NaCl (0.5-5%) at 20 ^{o}C; **C.** The effects of metal ions on the enzyme activity. Assay mixtures contained 50 mM Tris-HCl (pH 8.0), 2.5 μM NADH, 0.02 mg protein and 200 mg/L phenol at 20 ^{o}C with 1 mM of each metal ions; **D.** The effects of temperature on the enzyme activity. Assay mixtures contained 50 mM Tris-HCl (pH 8.0), 2.5 μM NADH, 0.02 mg protein and 200 mg/L phenol at different temperature (20-70 ^{o}C).
