# Supplementary material for: Characterization of a Novel Phenol Hydroxylase in Indoles Biotranformation from a Strain Arthrobacter sp. W1
Source: PLoS One. 2012 Sep 13;7(9):e44313. doi: 10.1371/journal.pone.0044313 (PMC3441600; doi:10.1371/journal.pone.0044313)
Supplement: Figure S2 — Gene cluster analysis and primary structure alignment of PHN component. A. PH gene cluster from strain W1 and related strains Comamonas testosteroni R5, Ralstoniaeutropha E2, Pseudomonas sp. CF600, Pseudomonas putida H. B. Structural alignment of primary structure of PHNs from Pseudomonas sp. OX1, Pseudomonas sp. CF600, Acinetobacter radioresistens S13, Arthrobacter sp. W1, Ralstonia eutropha E2 and Bacillus thermoleovorans sp. A2. The top line shows the secondary structure of PHN of Pseudomonas sp. OX1. The conserved residues are shown in red background. (PDF) [file pone.0044313.s002.pdf]

A.

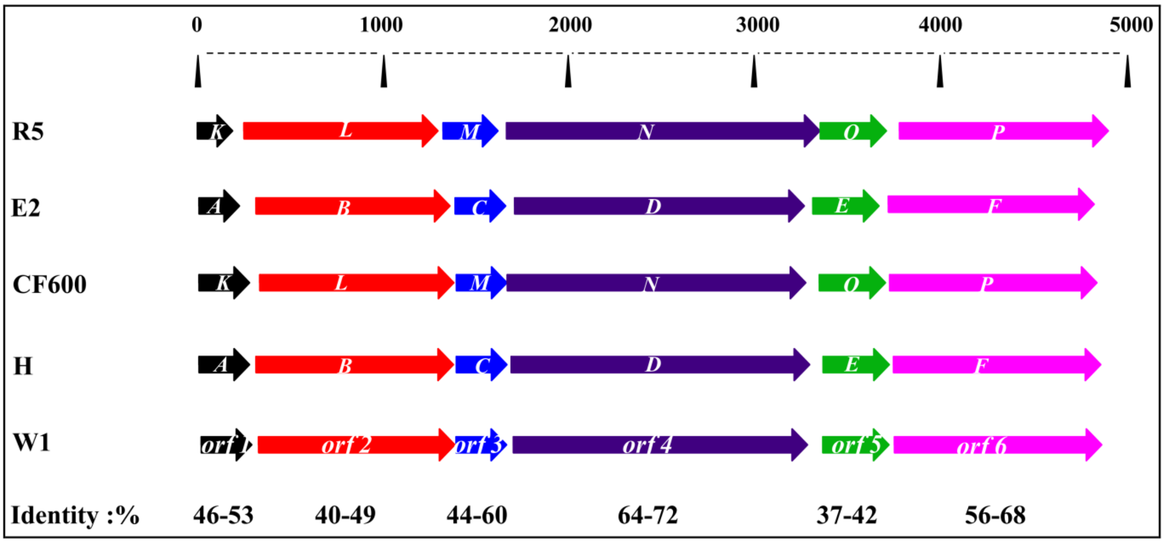

B.

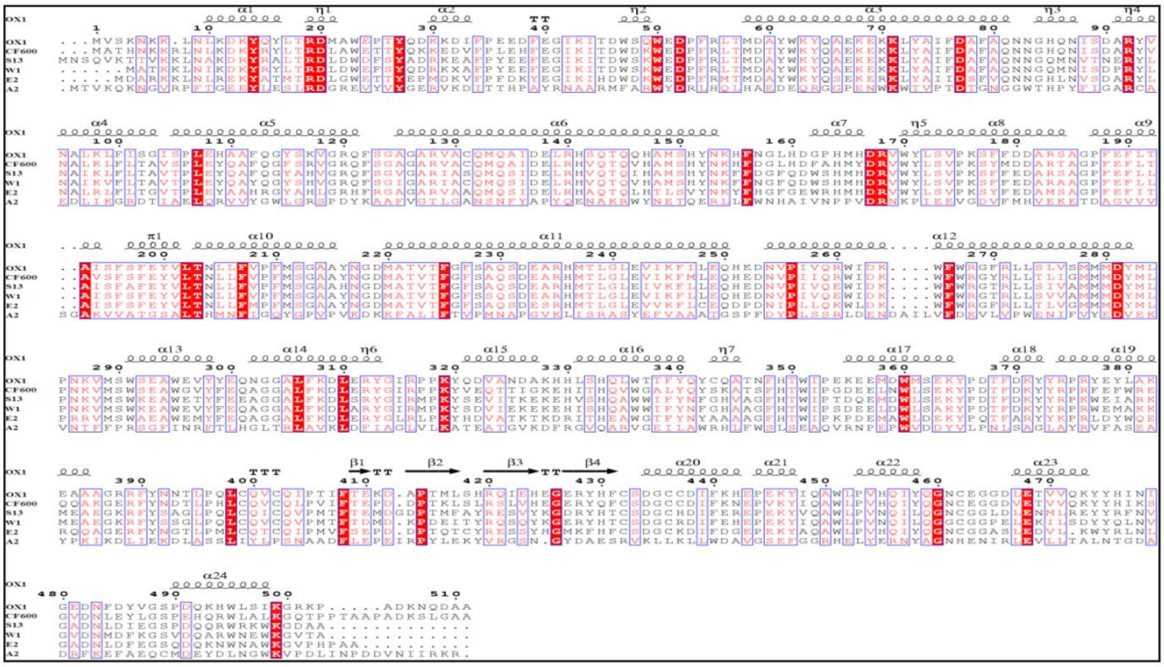

Figure S2. Gene cluster analysis and primary structure alignment of PHN component. **A.** PH gene cluster from strain W1 and related strains *Comamonas testosteroni* R5, *Ralstoniaeutropha* E2, *Pseudomonas* sp. CF600, *Pseudomonas putida* H. **B.** Structural alignment of primary structure of PHNs from *Pseudomonas* sp. OX1, *Pseudomonas* sp. CF600, *Acinetobacter radioresistens* S13, *Arthrobacter* sp. W1, *Ralstonia eutropha* E2 and *Bacillus thermoleovorans* sp. A2. The top line shows the secondary structure of PHN of *Pseudomonas* sp. OX1. The conserved residues are shown in red background.
