# Supplementary material for: Characterization of a Novel Phenol Hydroxylase in Indoles Biotranformation from a Strain Arthrobacter sp. W1
Source: PLoS One. 2012 Sep 13;7(9):e44313. doi: 10.1371/journal.pone.0044313 (PMC3441600; doi:10.1371/journal.pone.0044313)
Supplement: Figure S3 — Mass spectra of purified indigoid products formed from indoles by strain PH_IND. A. Products of indole transformation; B. Products of 4-methylindole transformation; C. Products of 5-methylindole transformation; D. Products of 7-methylindole transformation; E. Products of 4-chloroindole transformation; F. Products of 7-chloroindole transformation; G. Products of 5-methoxyindole transformation. HPLC-MS conditions were as follows: HPLC, 65% (v/v) CH3OH (in H2O containing 0.1% formic acid) for 10 min, followed by a 65–75% (v/v) CH3OH linear gradient over 20 min; MS was equipped with a standard API-1 atmospheric pressure chemical ionization (APCI) source in the positive or negative ion mode. N2 was used as a sheath gas (50 p.s.i.), vaporizer temperature was set to 350°C, and the corona current was maintained at 5 µA. The capillary was set at 220°C and 25 V (or −25 V in the negative mode). The tube lens voltage was set to 80 V (or to −96 V in the negative mode). The collision-induced dissociation was set to −30 V in tandem mass spectrometry experiments. (PDF) [file pone.0044313.s003.pdf]

A.

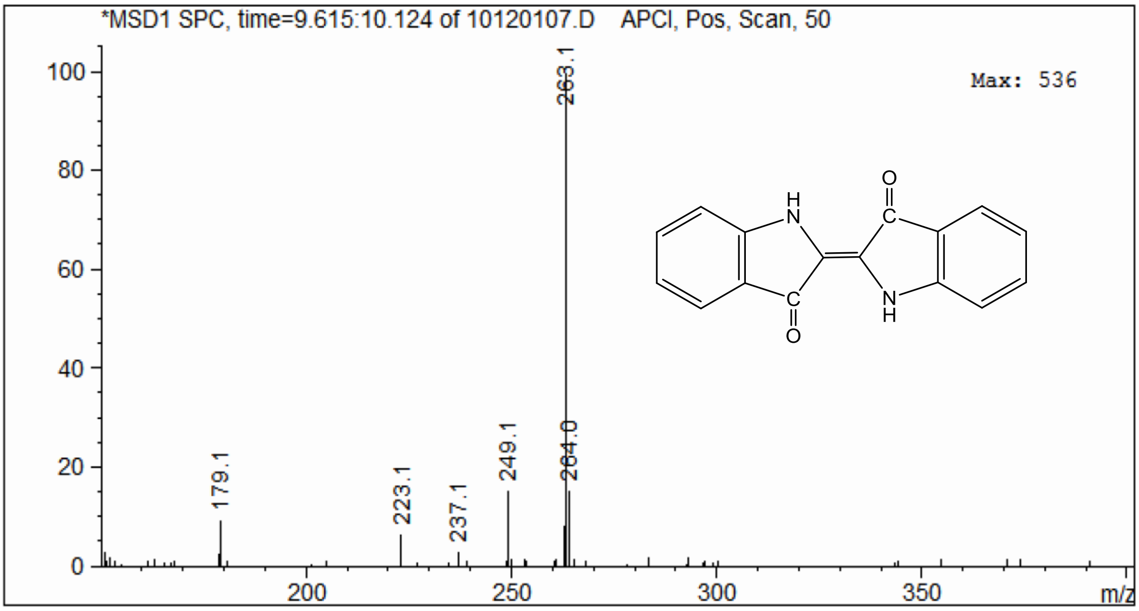
*MSD1 SPC, time=9.615:10.124 of 10120107.D APCI, Pos, Scan, 50
Max: 536
100
80
60
40
20
0
263.1
179.1
223.1
237.1
249.1
264.0
200
250
300
350
m/z
H
N
O
C
C
O
N
H

B.

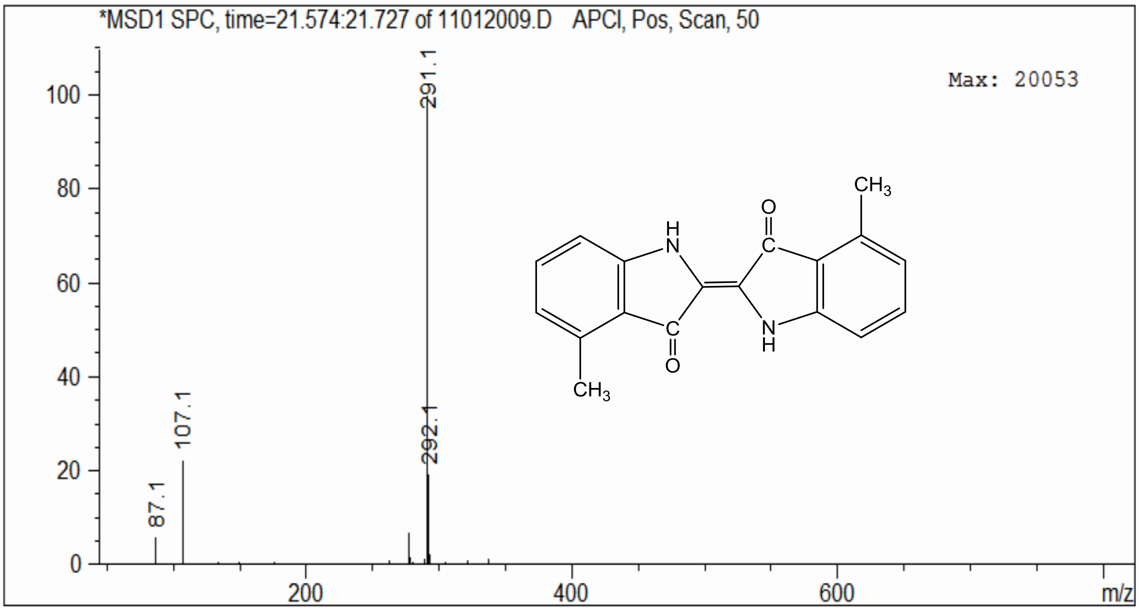
*MSD1 SPC, time=21.574:21.727 of 11012009.D APCI, Pos, Scan, 50
Max: 20053
100
80
60
40
20
0
291.1
107.1
87.1
292.1
200
400
600
m/z
CH3
O
H
N
C
C
O
N
H
CH3

C.

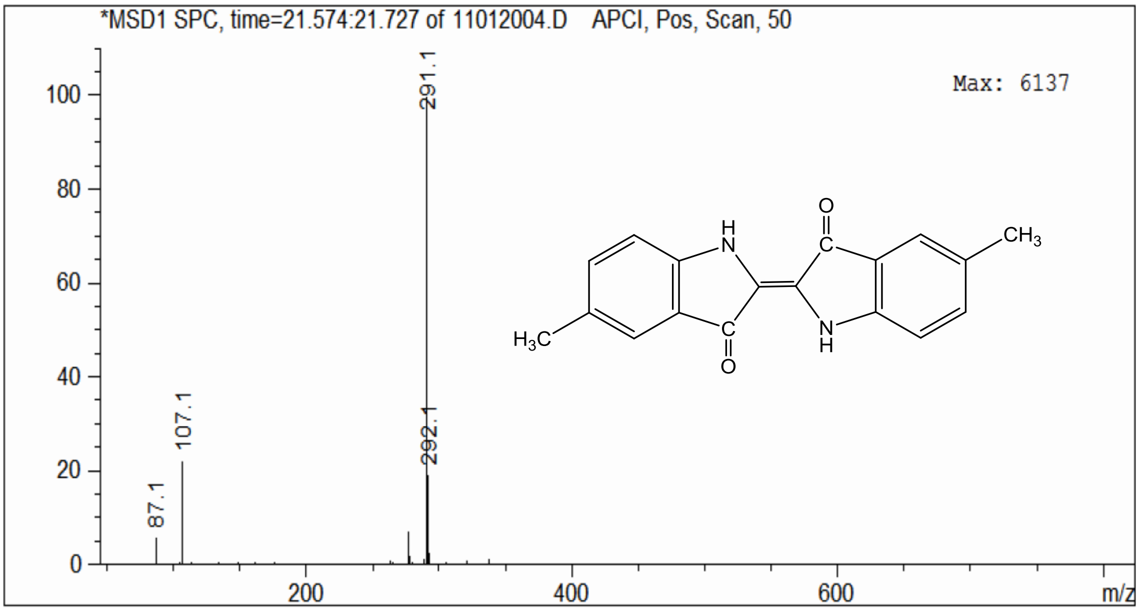
*MSD1 SPC, time=21.574:21.727 of 11012004.D APCI, Pos, Scan, 50
Max: 6137
291.1
292.1
107.1
87.1
100
80
60
40
20
0
200
400
600
m/z
H
N
O
C
CH3
H3C
C
O
N
H

D.

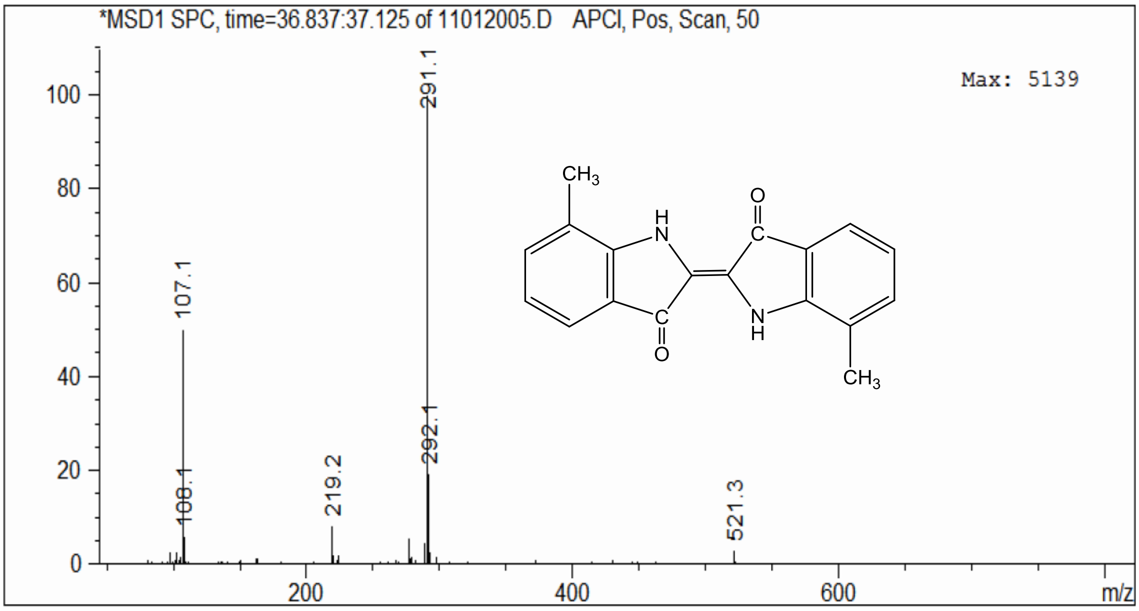
*MSD1 SPC, time=36.837:37.125 of 11012005.D APCI, Pos, Scan, 50
Max: 5139
291.1
107.1
292.1
219.2
108.1
521.3
100
80
60
40
20
0
200
400
600
m/z
CH3
H
N
O
C
C
O
N
H
CH3

E.

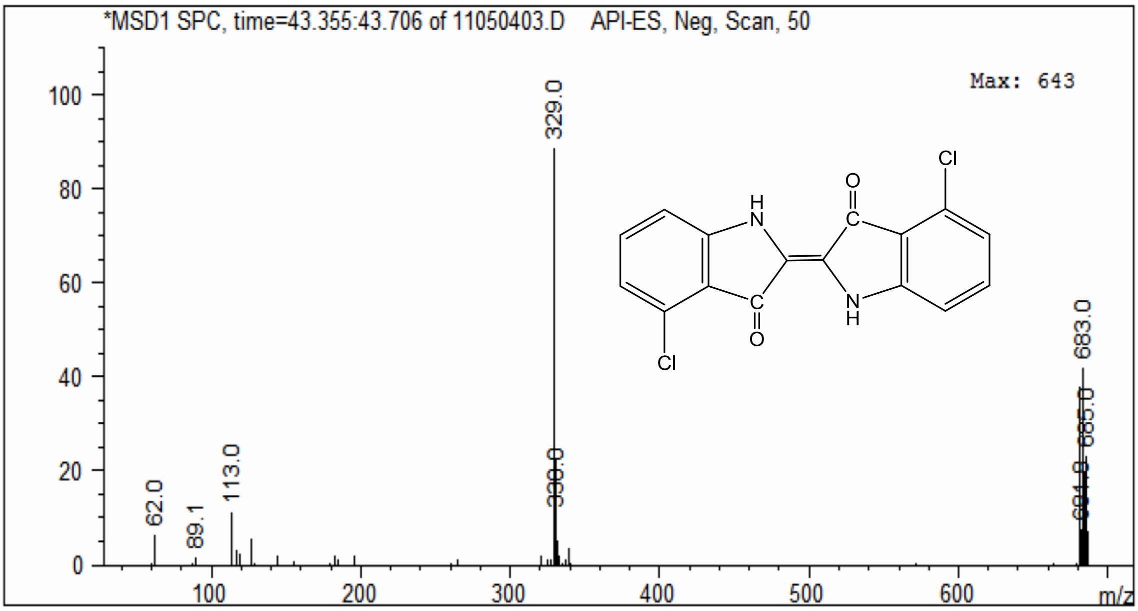
*MSD1 SPC, time=43.355:43.706 of 11050403.D API-ES, Neg, Scan, 50
Max: 643
100
80
60
40
20
0
62.0
89.1
113.0
329.0
683.0
100
200
300
400
500
600
m/z
Cl
O
C
H
N
N
H
C
O
Cl

F.

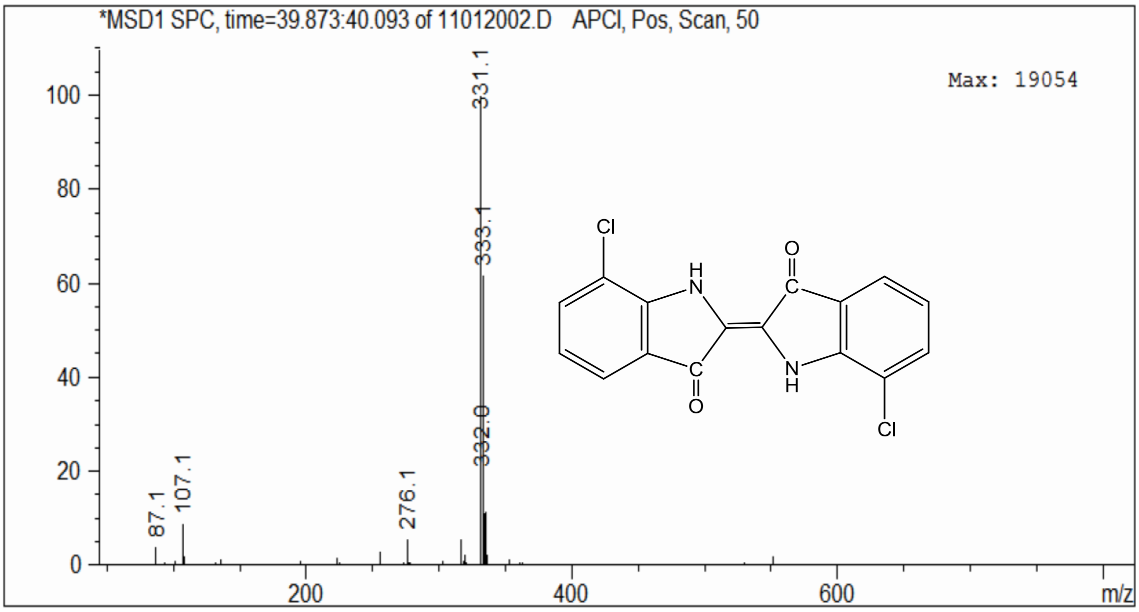
*MSD1 SPC, time=39.873:40.093 of 11012002.D APCI, Pos, Scan, 50
Max: 19054
100
80
60
40
20
0
87.1
107.1
276.1
331.1
333.1
332.0
200
400
600
m/z
Cl
H
N
O
C
C
O
N
H
Cl

G.

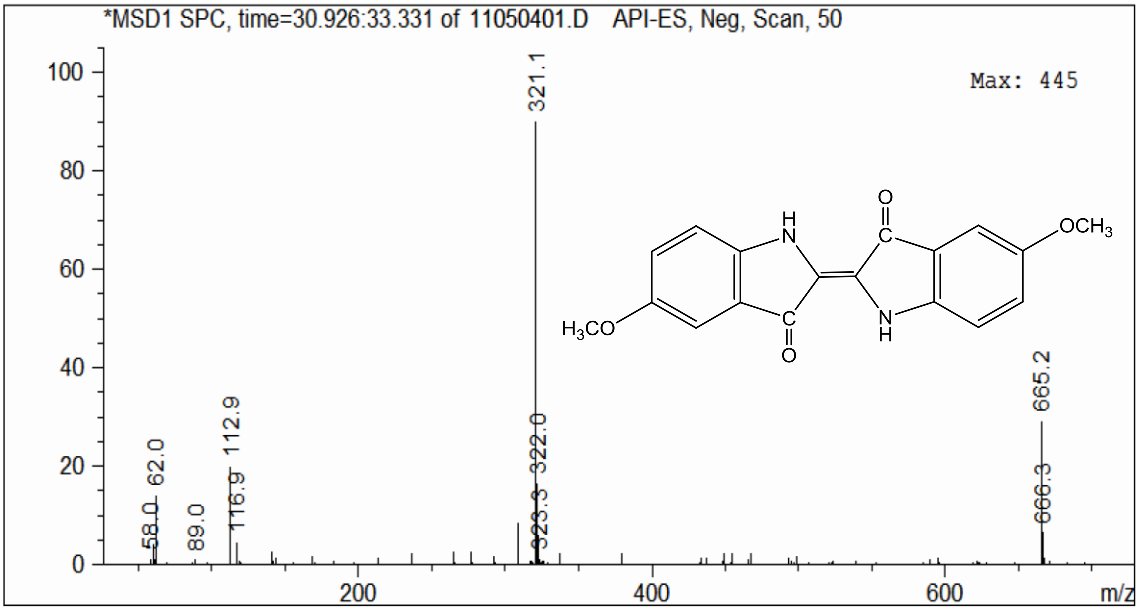

Figure S3. Mass spectra of purified indigoid products formed from indoles by strain PH_$_{\mathbf{IND}}$. A. Products of indole transformation; **B.** Products of 4-methylindole transformation; **C.** Products of 5-methylindole transformation; **D.** Products of 7-methylindole transformation; **E.** Products of 4-chloroindole transformation; **F.** Products of 7-chloroindole transformation; **G.** Products of 5-methoxyindole transformation. HPLC-MS conditions were as follows: HPLC, 65% (v/v) CH_3OH (in H_2O containing 0.1% formic acid) for 10 min, followed by a 65-75% (v/v) CH_3OH linear gradient over 20 min; MS was equipped with a standard API-1 atmospheric pressure chemical ionization (APCI) source in the positive or negative ion mode. N_2 was used as a sheath gas (50 p.s.i.), vaporizer temperature was set to 350 ^{o}C, and the corona current was maintained at 5 μA. The capillary was set at 220 ^{o}C and 25 V (or -25 V in the negative mode). The tube lens voltage was set to 80 V (or to -96 V in the negative mode). The collision-induced dissociation was set to -30 V in tandem mass spectrometry experiments.
