# Supplementary material for: Characterization of a Novel Phenol Hydroxylase in Indoles Biotranformation from a Strain Arthrobacter sp. W1
Source: PLoS One. 2012 Sep 13;7(9):e44313. doi: 10.1371/journal.pone.0044313 (PMC3441600; doi:10.1371/journal.pone.0044313)
Supplement: Figure S4 — 1H NMR spectra of purified indigoid products formed from indoles by strain PH_IND. A. Products of indole transformation; B. Products of 4-methylindole transformation; C. Products of 5-methylindole transformation; D. Products of 7-methylindole transformation; E. Products of 4-chloroindole transformation; F. Products of 7-chloroindole transformation; G. Products of 5-methoxyindole transformation. 1H NMR was carried out at 298 K with Bruker Avance II 400 M and 400 MHz instrument and the samples were dissolved in d −6 Me2SO. (PDF) [file pone.0044313.s004.pdf]

A.

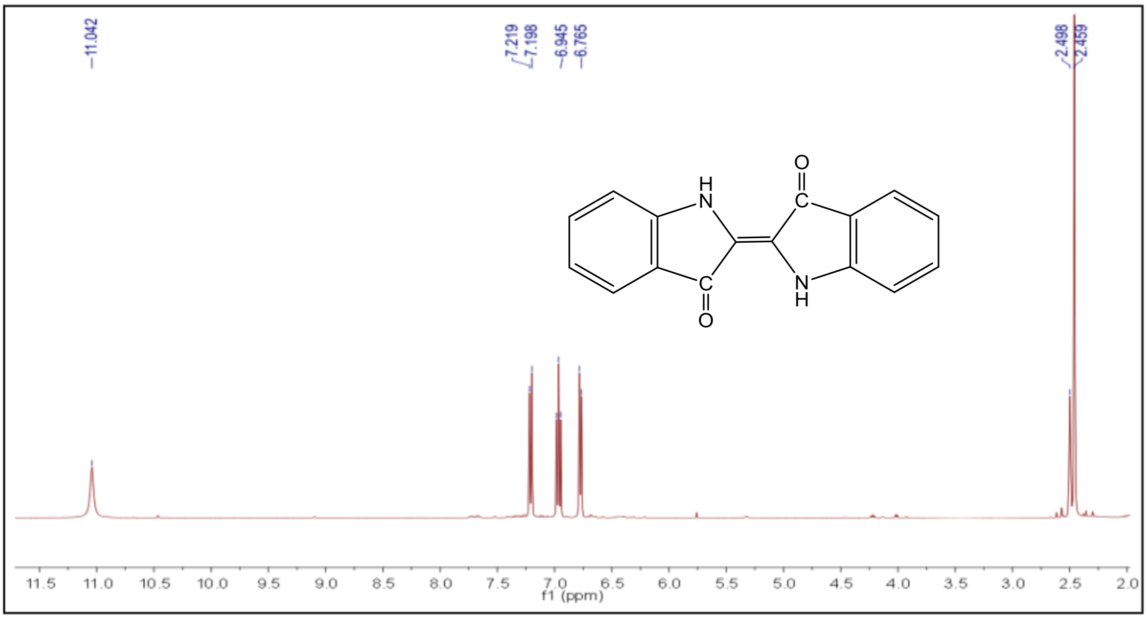
11.042
7.219
7.198
6.945
6.765
2.498
2.459
f1 (ppm)

B.

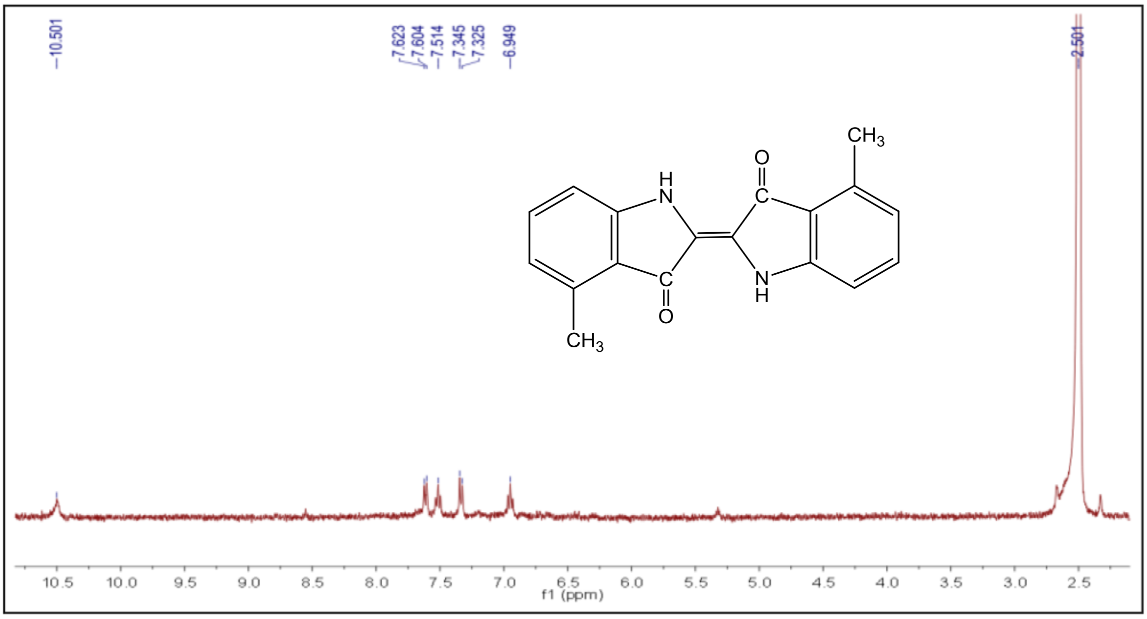
10.501
7.623
7.604
7.514
7.345
7.325
6.949
2.501
f1 (ppm)

C.

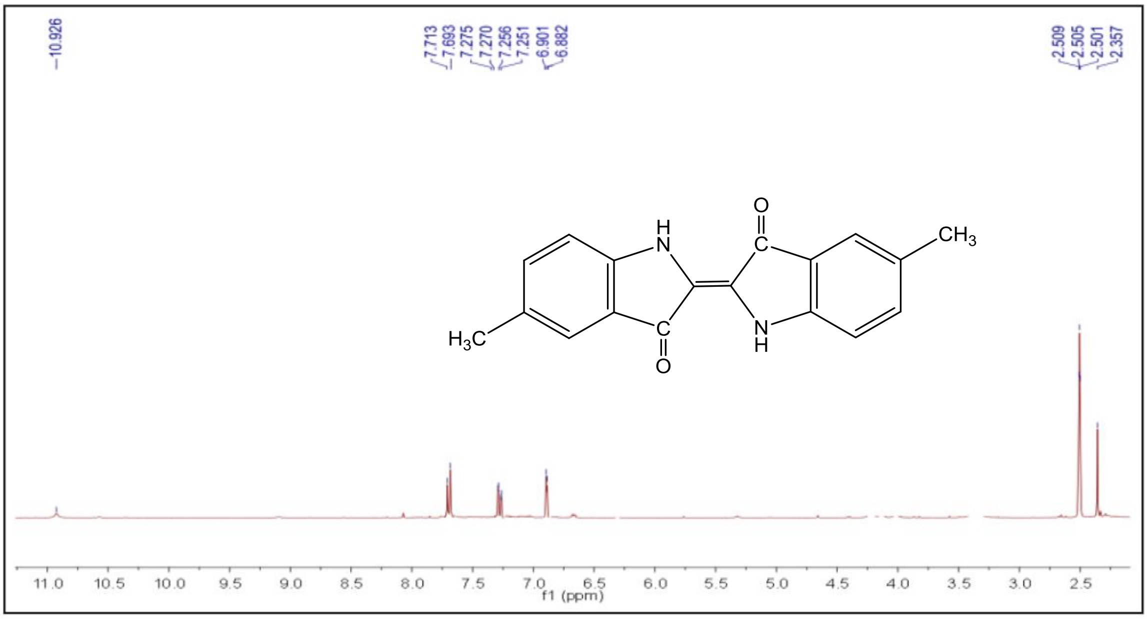
10.926
7.713
7.693
7.275
7.270
7.256
7.251
6.901
6.882
2.509
2.505
2.501
2.357
f1 (ppm)

D.

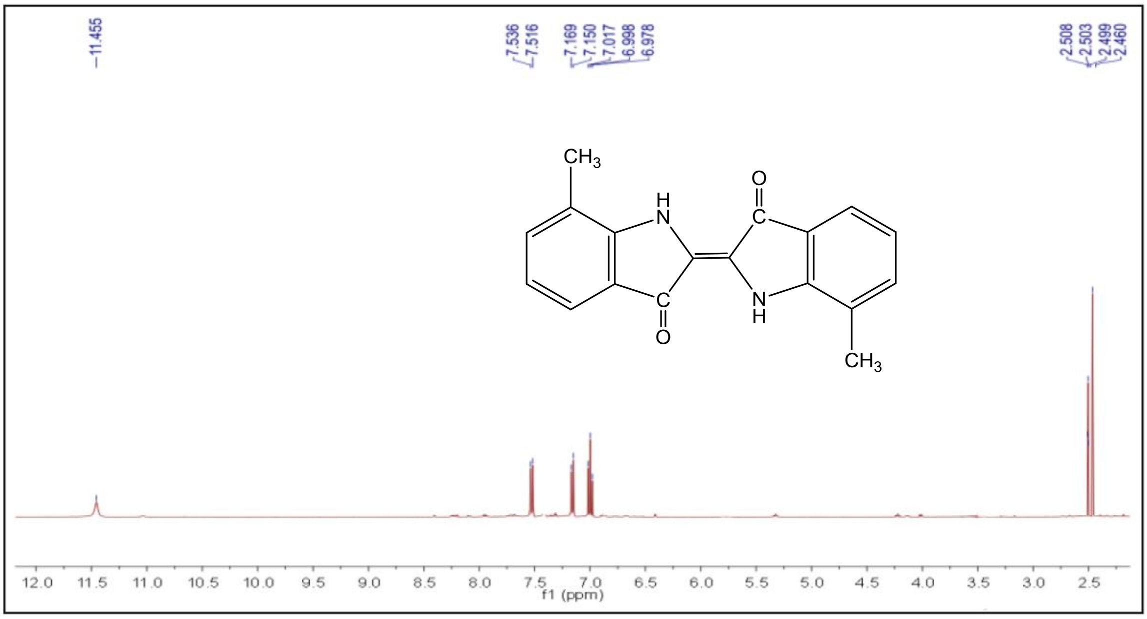
11.455
7.536
7.516
7.169
7.150
7.017
6.998
6.978
2.508
2.503
2.499
2.460
f1 (ppm)

E.

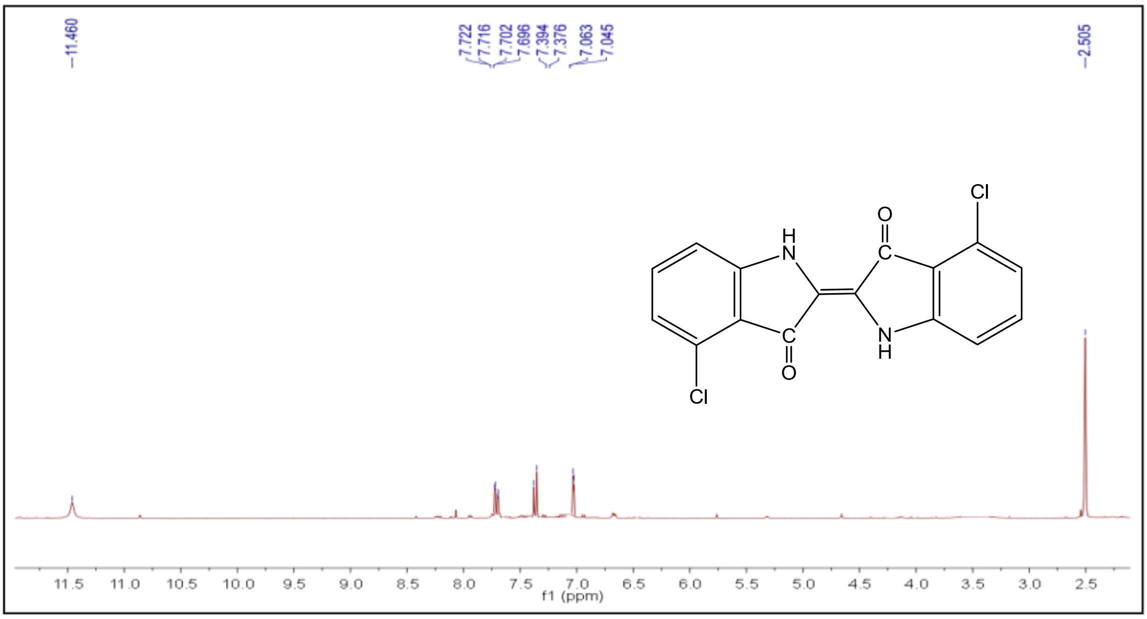

11.460
7.722
7.716
7.702
7.696
7.394
7.376
7.063
7.045
2.505
Cl
O
H
N
C
C
N
H
O
Cl
11.5 11.0 10.5 10.0 9.5 9.0 8.5 8.0 7.5 7.0 6.5 6.0 5.5 5.0 4.5 4.0 3.5 3.0 2.5
f1 (ppm)

F.

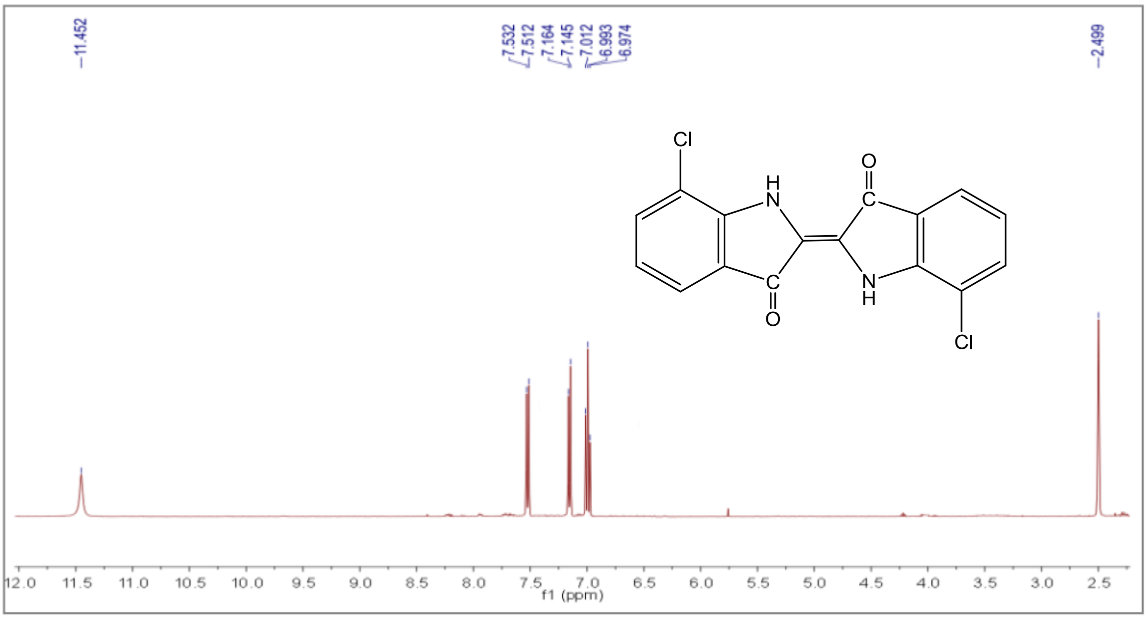

11.452
7.532
7.512
7.164
7.145
7.012
6.993
6.974
2.499
Cl
H
N
O
C
C
N
H
O
Cl
12.0 11.5 11.0 10.5 10.0 9.5 9.0 8.5 8.0 7.5 7.0 6.5 6.0 5.5 5.0 4.5 4.0 3.5 3.0 2.5
f1 (ppm)

G.

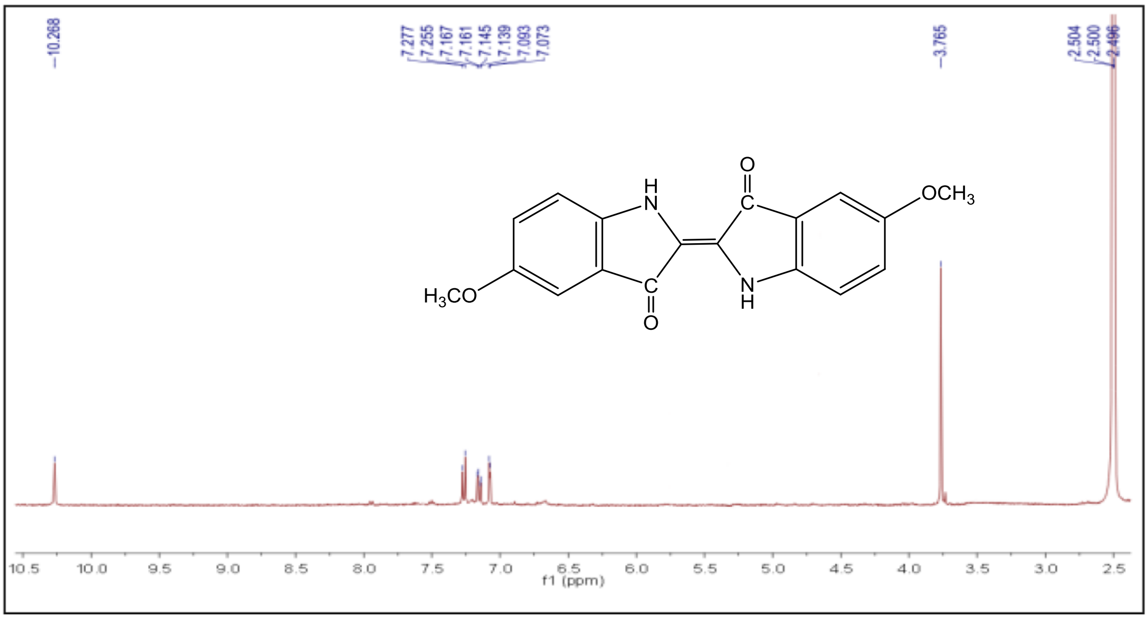

Figure S4. ^{1}H NMR spectra of purified indigoid products formed from indoles by strain PH_{IND}. A. Products of indole transformation; **B.** Products of 4-methylindole transformation; **C.** Products of 5-methylindole transformation; **D.** Products of 7-methylindole transformation; **E.** Products of 4-chloroindole transformation; **F.** Products of 7-chloroindole transformation; **G.** Products of 5-methoxyindole transformation. ^{1}H NMR was carried out at 298 K with Bruker Avance II 400M and 400 MHz instrument and the samples were dissolved in d_{-6} Me_2SO.
