# Supplementary material for: Characterization of a Novel Phenol Hydroxylase in Indoles Biotranformation from a Strain Arthrobacter sp. W1
Source: PLoS One. 2012 Sep 13;7(9):e44313. doi: 10.1371/journal.pone.0044313 (PMC3441600; doi:10.1371/journal.pone.0044313)
Supplement: Figure S5 — Identification of new purple product derived from indole. A. Mass spectra; B. 1H NMR spectra; C. 13C NMR spectra; D. 1H-1H COSY; E. 1H-13C HSQC; F. 1H-13C HMBC. The conditions for each spectrum were the same with those described above. (PDF) [file pone.0044313.s005.pdf]

A.

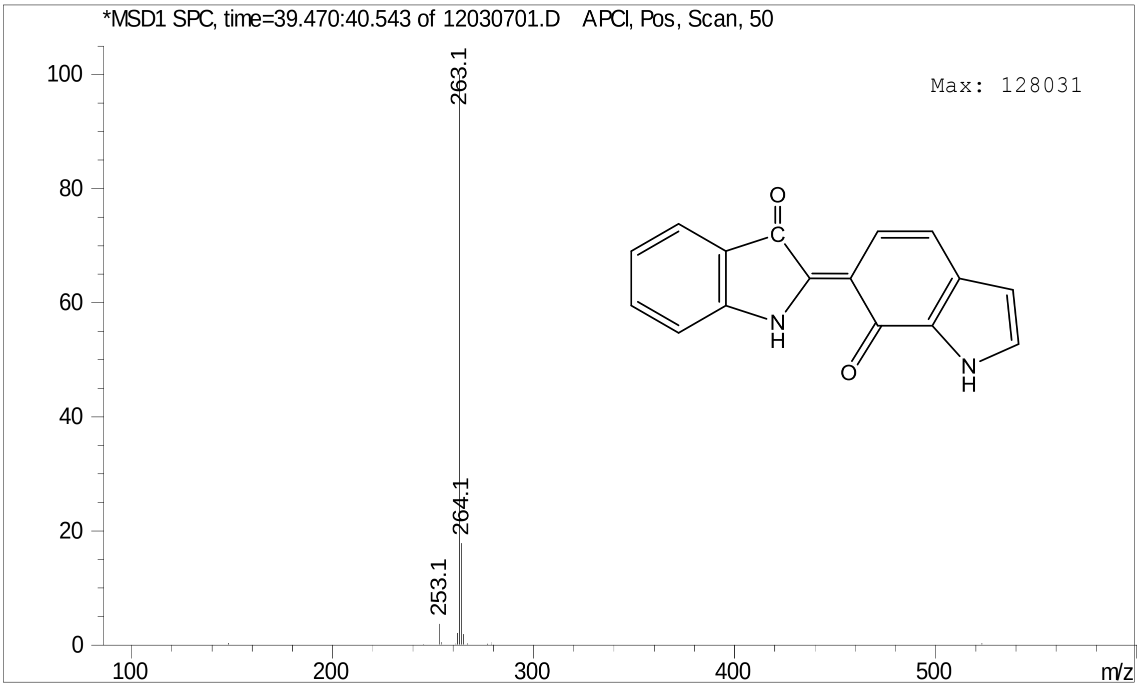
*MSD1 SPC, time=39.470:40.543 of 12030701.D APCI, Pos, Scan, 50
Max: 128031
263.1
264.1
253.1
100
80
60
40
20
0
100
200
300
400
500
m/z
O
C
N
H
O
N
H

B.

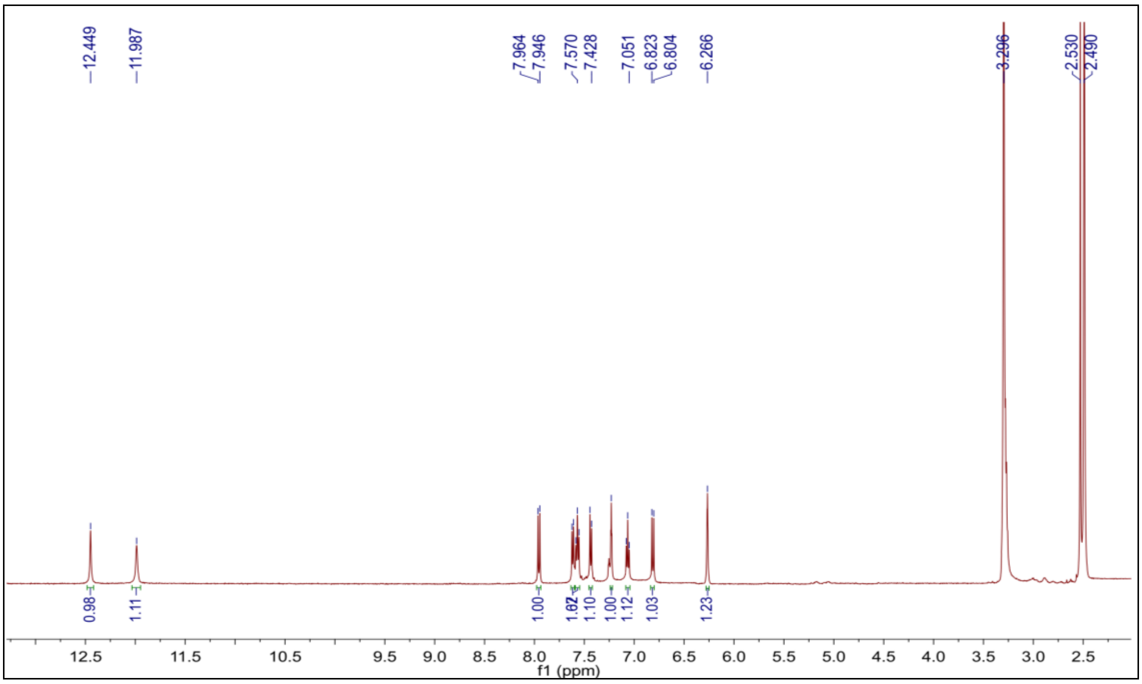
12.449
11.987
7.964
7.946
7.570
7.428
7.051
6.823
6.804
6.266
3.296
2.530
2.490
0.98
1.11
1.00
1.02
1.10
1.00
1.12
1.03
1.23
12.5
11.5
10.5
9.5
9.0
8.5
8.0
7.5
7.0
6.5
6.0
5.5
5.0
4.5
4.0
3.5
3.0
2.5
f1 (ppm)

C.

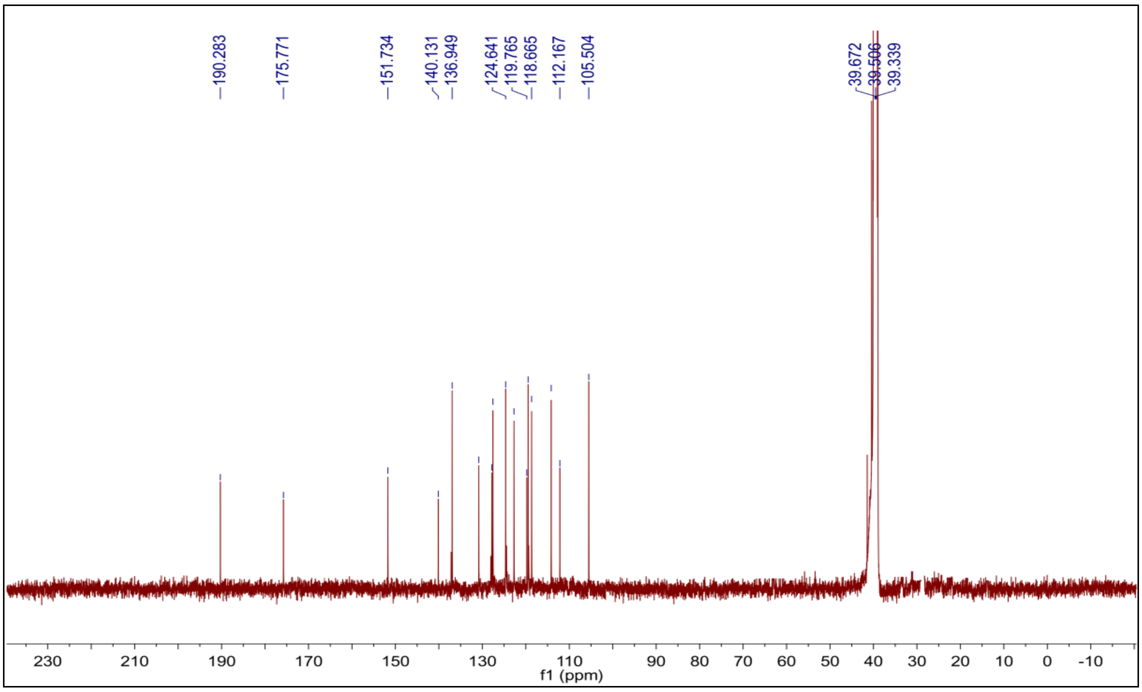
190.283
175.771
151.734
140.131
136.949
124.641
119.765
118.665
112.167
105.504
39.672
39.506
39.339
230
210
190
170
150
130
110
90
80
70
60
50
40
30
20
10
0
-10
f1 (ppm)

D.

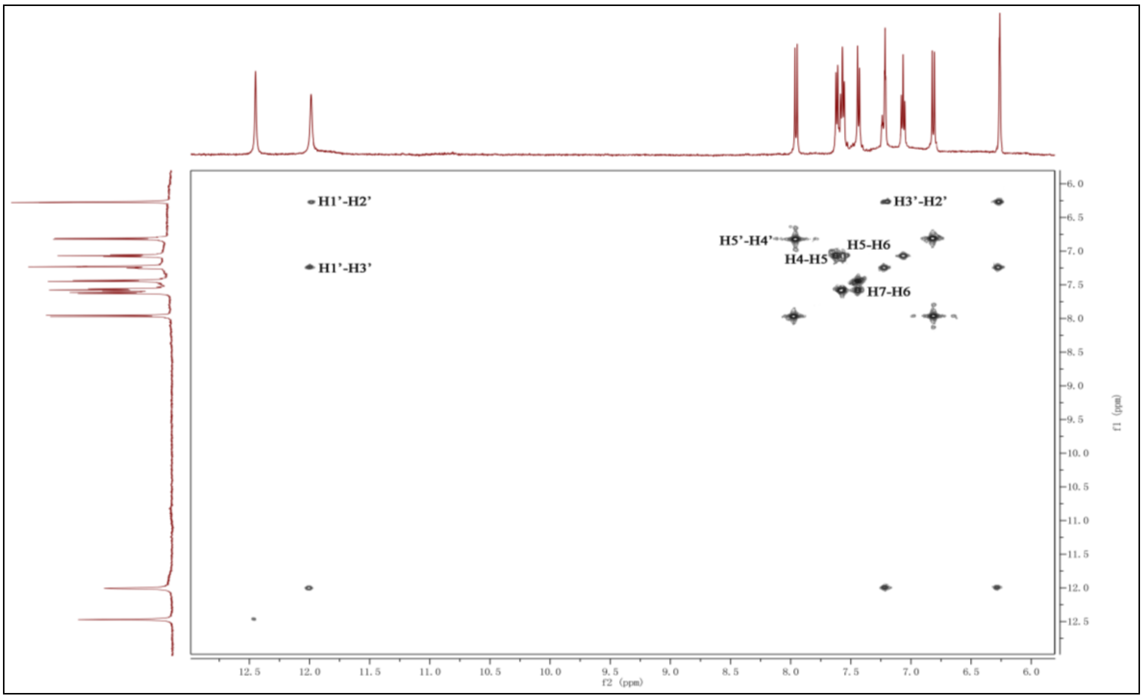
H1'-H2'
H1'-H3'
H3'-H2'
H5'-H4'
H5-H6
H4-H5
H7-H6
f2 (ppm)
f1 (ppm)

E.

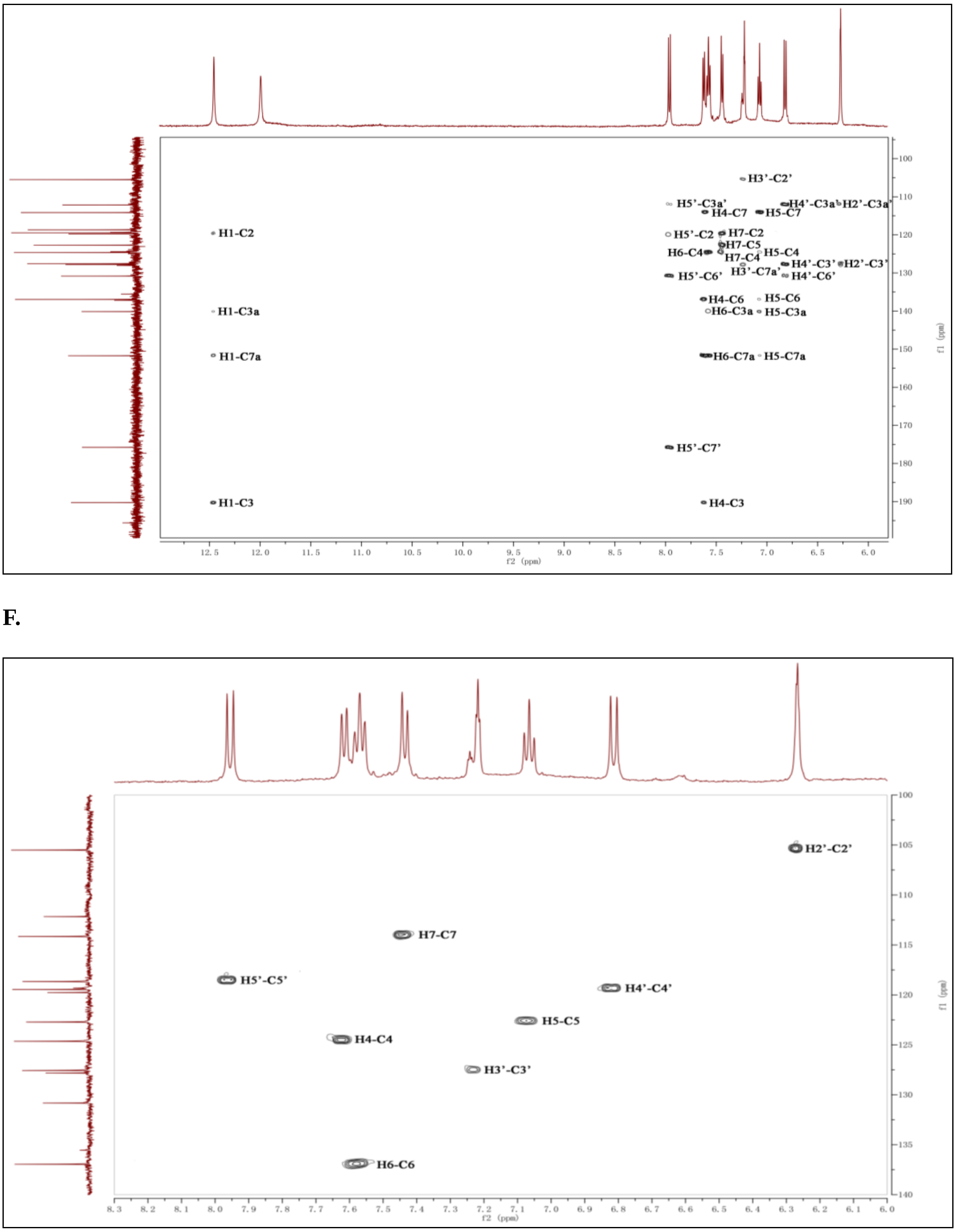

F.

Figure S5. Identification of new purple product derived from indole. A. Mass spectra; **B.** ^{1}H NMR spectra; **C.** ^{13}C NMR spectra; **D.** ^{1}H-^{1}H COSY; **E.** ^{1}H-^{13}C HSQC; **F.** ^{1}H-^{13}C HMBC. The conditions for each spectrum were the same with those described above.
