# Supplementary material for: Characterization of a Novel Phenol Hydroxylase in Indoles Biotranformation from a Strain Arthrobacter sp. W1
Source: PLoS One. 2012 Sep 13;7(9):e44313. doi: 10.1371/journal.pone.0044313 (PMC3441600; doi:10.1371/journal.pone.0044313)
Supplement: Figure S6 — SDS-PAGE analysis of protein samples of strain PH_IND and its mutants. Line 1. Cell extracts of strain PH_IND; Line 2. Cell extracts of strain PH_IND-Asn-202; Line 3. Cell extracts of strain PH_IND-His-139; M. Protein markers. Arrows show the positions of the six ORFs. ORF1. 10.4 kDa; ORF2. 37.6 kDa; ORF3. 10.5 kDa; ORF4. 59.2 kDa; ORF5. 13.5 kDa; ORF6. 38.6 kDa. SDS-PAGE was performed on 5% and 15% acrylamide concentrations for the concentrating and separating gels, respectively. (PDF) [file pone.0044313.s006.pdf]

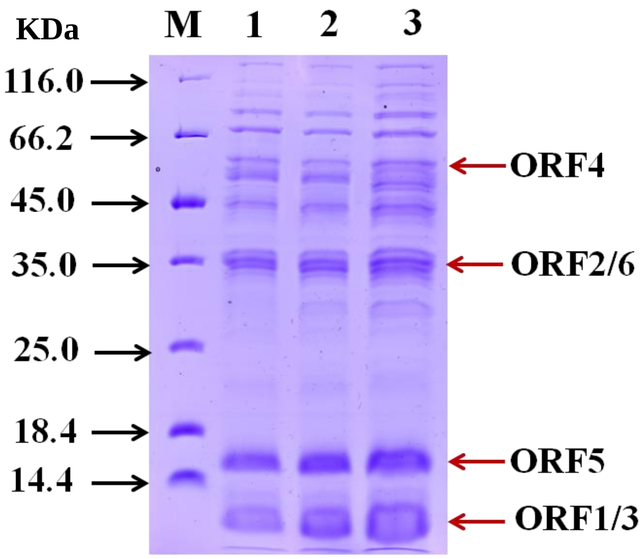


**Figure S6. SDS-PAGE analysis of protein samples of strain PH_$_{IND}$ and its mutants. Line 1.** Cell extracts of strain PH_$_{IND}$; **Line 2.** Cell extracts of strain PH_$_{IND}$-Asn-202; **Line 3.** Cell extracts of strain PH_$_{IND}$-His-139; **M.** Protein markers. Arrows show the positions of the six ORFs. **ORF1.** 10.4 kDa; **ORF2.** 37.6 kDa; **ORF3.** 10.5kDa; **ORF4.** 59.2 kDa; **ORF5.** 13.5 kDa; **ORF6.** 38.6 kDa. SDS-PAGE was performed on 5% and 15% acrylamide concentrations for the concentrating and separating gels, respectively.
