# Supplementary material for: Characterization of a Novel Phenol Hydroxylase in Indoles Biotranformation from a Strain Arthrobacter sp. W1
Source: PLoS One. 2012 Sep 13;7(9):e44313. doi: 10.1371/journal.pone.0044313 (PMC3441600; doi:10.1371/journal.pone.0044313)
Supplement: Figure S7 — Biotransformation of indole by strain PH_IND and its mutans. A. Indole biotransformation by strain PH_IND; B. Indole biotransformation by strain PH_IND-Asn-202; C. Indole biotransformation by strain PH_IND-His-139. The left bottles were the control groups at 0 h; the right bottles were the test groups at 12 h. (PDF) [file pone.0044313.s007.pdf]

A. **B.** **C.**

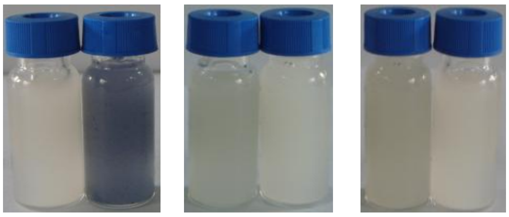

Figure S7. Biotransformation of indole by strain PH_$_{IND}$ and its mutans. A. Indole biotransformation by strain PH_$_{IND}$; **B.** Indole biotransformation by strain PH_$_{IND}$-Asn-202; **C.** Indole biotransformation by strain PH_$_{IND}$-His-139. The left bottles were the control groups at 0 h; the right bottles were the test groups at 12 h.
