# Supplementary material for: Characterization of a Novel Phenol Hydroxylase in Indoles Biotranformation from a Strain Arthrobacter sp. W1
Source: PLoS One. 2012 Sep 13;7(9):e44313. doi: 10.1371/journal.pone.0044313 (PMC3441600; doi:10.1371/journal.pone.0044313)
Supplement: Table S1 — Amino acid identity between PHW1 and other binuclear iron hydroxylases. (PDF) [file pone.0044313.s008.pdf]

Table S1. Amino acid identity between PH_{W1} and other binuclear iron hydroxylases

ORF (gene)	Size (aa)	Position of start-stop codon	Gene product description	Organism	Accession No.	Identity (%)
ORF1	90	181-453	phenol hydroxylase component	*Acinetobacter* sp. PHEA-2	ABS81292	52
(*K*)			conserved hypothetical protein	*Acinetobacter* sp. SH024	EFF87579	52
			phenol 2-monooxygenase P0 component	*Pseudomonas* sp. CF600	AAA25939	53
ORF2	335	477-1484	phenol hydroxylase P1 protein	*Acinetobacter* sp. SK82	EET83667	60
(*L*)			phenol 2-monooxygenase	*Acinetobacter* sp. DR1	ADI91547	61
			phenol hydroxylase component phL	*Pseudomonas* sp. OX1	AAO47356	49
ORF3	90	1484-1756	phenol hydroxylase component	*Acinetobacter calcoaceticus*	CAA85382	70
(*M*)			phenol hydroxylase component	*Ralstonia* sp. E2	AAC32454	51
			DMS oxygenase component	*Acinetobacter* sp.	BAA23332	70
ORF4	501	1775-3280	phenol hydroxylase component	*Acinetobacter* sp. RUH2624	EEX00476	87
(*N*)			phenol hydroxylase oxygenase component	*Acinetobacter* sp. MO	ACL31145	86
			methane/phenol/toluene hydroxylase	*Acinetobacter* sp. DR1	ADI9154	87
ORF5	120	3347-3709	phenol hydroxylase component	*Acinetobacter* sp. MO	ACL31146	54
(*O*)			phenol hydroxylase component gamma subunit	*Acinetobacter* sp. G16	ACS74439	54
			phenol hydroxylase P4 protein	*Acinetobacter*sp. SK82	EET83670	54
ORF6	353	3725-4786	DMS oxygenase component	*Acinetobacter* sp.	BAA23335	72
(*P*)			phenol hydroxylase, Ferredoxin subunit	*Acinetobacter* sp. DR1	ADI91551	73
			phenol hydroxylase component	*Acinetobacter calcoaceticus*	CAD92316	73
