# Supplementary material for: Characterization of a Novel Phenol Hydroxylase in Indoles Biotranformation from a Strain Arthrobacter sp. W1
Source: PLoS One. 2012 Sep 13;7(9):e44313. doi: 10.1371/journal.pone.0044313 (PMC3441600; doi:10.1371/journal.pone.0044313)
Supplement: Table S2 — Production of dyestuffs from indole derivatives by Escherichia coli expressing different oxygenases. (PDF) [file pone.0044313.s009.pdf]

Table S2. Production of dyestuffs from indole derivatives by *Escherichia coli* expressing different oxygenases

Indole derivatives	PH_$_{IND}$*	mPH$_{KL28}$*	mPH$_{KL33}$*	NDO*	TDO*	P450 2A6 L240C/N297Q*	P4502A6 N297Q/I300V*	P4502A6 mutants*	2-hydroxybiphenyl3-monooxygenase*
Indole	Blue	Blue	-	Blue	Blue	Blue	Blue	Blue	Blue
2-methylindole	Yellow	-	-	-	-				
3-methylindole	-	-	-	-	-				
4-methylindole	Blue	Blue	-	Blue	-			Blue	
5-methylindole	Blue	Blue	-	Blue	-	Blue			
7-methylindole	Blue	Blue	-	Blue	-			Blue	
5-methoxyindoe	Blue	Blue	Blue	-	-	Blue		Green	
5-bromoindole	Blue	Blue	-	Blue	-				Pink
5-aminoindole	-	-	-	-	-				
4-hydroxyindole	Blue	Purple	-	-	-				Purple
4-chloroindole	Blue	-	-	-	-		Blue	Blue	
5-chloroindole	Blue	Blue	-	Blue	-				
6-chloroindole	Pink	Pink	-	Pink	Pink				
7-chloroindole	Purple	Purple	-	Purple	Purple				
5-nitroindole	Pink	Pink	-	Pink	Pink			Purple	
6-nitroindole	Green	Green	-	Green	-			Green	
Reference	This study	(6)	(6)	(7)	(7)	(8)	(12)	(36)	(20)

* PH_$_{IND}$ from *Arthrobacter* sp. W1; mPH$_{KL28}$ from *Pseudomonas* sp. KL28; mPH$_{KL33}$ form *Pseudomonas* sp. KL33; NDO from *Pseudomonas* sp. NCIB 9816-4; TDO from *Pseudomonas putida* F1; P450 2A6 L240C/N297Q of cytochrome P450 2A6; P450 2A6 N297Q/I300V of cytochrome P450 2A6; P450 2A6 mutants of cytochrome P450 2A6; 2-hydroxybiphenyl 3-monooxygenase from *Pseudomonas azelaica* HBP1.
