# Supplementary material for: Characterization of a Novel Phenol Hydroxylase in Indoles Biotranformation from a Strain Arthrobacter sp. W1
Source: PLoS One. 2012 Sep 13;7(9):e44313. doi: 10.1371/journal.pone.0044313 (PMC3441600; doi:10.1371/journal.pone.0044313)
Supplement: Table S3 — Oligonucleotide primers used in this study. (PDF) [file pone.0044313.s010.pdf]

**Table S3. Oligonucleotide primers used in this study**

| Primer | Sequence (5’to 3’) |
|---|---|
| Raw TEST-F | 5’- AGGCATCAAGATCACCGACTG -3’ |
| Raw TEST-R | 5’- CGCCAGAACCATTTATCGATC -3’ |
| SP1-5’ | 5’- GTCGTGCATATGGCTCCAGTCCTG -3’ |
| SP2-5’ | 5’- GGAGTAGCCCTGGTAAGCCTGGTA -3’ |
| SP3-5’ | 5’- ATATTCATCTGGCCATTGTTCTGCGC -3’ |
| SP1-5’-1 | 5’- ACGGTCAGTTGGCCCACACA -3’ |
| SP2-5’-1 | 5’- GCAGACCGATATAAACCTTGGAT -3’ |
| SP3-5’-1 | 5’- TGTAGGGCATGAAGGCGGCT -3’ |
| SP3-5’-2 | 5’- TCGTATGTCGCTTCCTGGTAG -3’ |
| SP1-3’ | 5’- CAGGACTGGAGCCATATGCACGAC -3’ |
| SP2-3’ | 5’- TCCTTCGAGTATGTGCTGACCAAC -3’ |
| SP3-3’ | 5’- AGTCTGACGAAGCACGCCACATGA -3’ |
| SP3-3’-1 | 5’- GCGACTACTACCAGCTGAACG -3’ |
| SP3-3’-2 | 5’- CCTGCCCGAATCCGATATGGTCA -3’ |
| SP3-3’-3 | 5’- AGATGGGGCAAGCGGCAAGGTG -3’ |
| TEST-F2 | 5’- TTGATGAAAACGGCGCTAAGCA -3’ |
| TEST-R | 5’- TCGTCTGCCCTTTTCATGGATGG -3’ |
| TEST-F | 5’- GCTGGAGTTCATGCTGGATTGTC -3’ |
| F | 5’-ATCGAGCTCATGTCAGACACGAGCTTGCCCACTTTGAC -3’ |
| R | 5’-CCCAAGCTTTTAAATACGCTTGAACAAGGCCGAGCGCACC-3’ |
| F02 | 5’- TGAGCGACTACTACCAGCTG -3’ |
| P2 | 5’- CACTTTGGTCGGTAACAGCC -3’ |
| P3 | 5’- CCCAAGCCTGTCTCTATGCC -3’ |
| P5 | 5’- TGACGCGTGATCTGGACTGG -3’ |
| P6 | 5’- TGTCCGTGCCCAAGTCTTTT -3’ |
| P10 | 5’- TTGCTGCCTCCGCCTCTTAT -3’ |
| P22 | 5’- ATATCGGTCTGCAAGACAAC -3’ |
| P25 | 5’- TGGAATAGATAAATAGGACG -3’ |
| BT-R02 | 5’- TCCTTTCCATTCATTCCAGCGCGCTT -3’ |
