# Supplementary material for: Characterization of a Novel Phenol Hydroxylase in Indoles Biotranformation from a Strain Arthrobacter sp. W1
Source: PLoS One. 2012 Sep 13;7(9):e44313. doi: 10.1371/journal.pone.0044313 (PMC3441600; doi:10.1371/journal.pone.0044313)
Supplement: Table S4 — Primers used for site-directed mutagenesis. (PDF) [file pone.0044313.s011.pdf]

Table S4. Primers used for site-directed mutagenesis

Mutants	Primers[*]
$PH_{\_IND}$-Asn-202	5'-AATT*GACGTC*AATTCAGCGGCATTGGCG-3'
	5'-GACGAGCTGCGT**<u>TTC</u>**GTCCAGACGCAG-3'
	5'-AACCTGCGTCTGGAC**<u>GAA</u>**ACGCAGCTC-3'
	5'-AATT*GTCGAC*ACCCACGTTCAGC-3'
$PH_{\_IND}$-His-139	5'-AATT*GACGTC*AATTCAGCGGCATTGGCG-3'
	5'-GCTCGCAGTCTG**<u>GCG</u>**AAGCACGCCACA-3'
	5'-TCATGTGGCGTGCTT**<u>CGC</u>**CAGACTGCG-3'
	5'-AATT*GTCGAC*ACCCACGTTCAGC-3'

[*] The italic characters indicate the introduction of restriction sites; the nucleotide changes are underlined and bold.
